# Structural elements of cyanobacterial co-factor-independent phosphoglycerate mutase that mediate regulation by PirC

**DOI:** 10.1128/mbio.03378-24

**Published:** 2025-04-03

**Authors:** Tim Orthwein, Janette T. Alford, Nathalie Sofie Becker, Phillipp Fink, Karl Forchhammer

**Affiliations:** 1 Interfaculty Institute of Microbiology and Infection Medicine Tübingen, University of Tübingen, Tübingen, Germany; Freie Universitat Berlin, Berlin, Germany

**Keywords:** PirC, PII, iPGAM, enzyme regulation, phosphogylcerate mutase, *Cyanobacteria*, *Synechocystis*

## Abstract

The 2,3-bisphosphoglycerate-independent phosphoglycerate mutase (iPGAM) has been identified as a regulating key point in the carbon storage metabolism of cyanobacteria. Upon nitrogen starvation, the iPGAM is inhibited by the P_II_-interacting regulator PirC, which is released from its interaction partner P_II_ due to elevated 2-oxoglutarate levels. *In silico* analysis of 338 different iPGAMs revealed a deep-rooted distinctive evolution of iPGAMs in cyanobacteria. Remarkably, cyanobacterial iPGAMs possess a unique loop structure and an extended C-terminus. Our mass photometry analysis suggests that iPGAM forms a complex with three individual PirC monomers. Biolayer interferometry revealed that the PirC–iPGAM complex is affected by the unique loop and the C-terminal structural elements of iPGAM. A C-terminally truncated iPGAM enzyme showed loss of control by PirC and twofold increased enzymatic activity compared to the iPGAM-WT (wild type), as demonstrated by enzyme assays. By contrast, deleting the loop structure significantly reduced the activity of this variant. Physiological experiments were carried out with different iPGAM variant strains of *Synechocystis*, in which these structural elements were deleted. The strain expressing the C-terminally truncated iPGAM showed a similar overproduction of polyhydroxybutyrate as deletion of the iPGAM regulator PirC. However, in contrast to the latter, these strains showed higher overall biomass accumulation, making them a better chassis for the production of polyhydroxybutyrate or other valuable substances than the PirC-deficient mutant.

**IMPORTANCE:**

The primordial cyanobacteria were responsible for developing oxygenic photosynthesis early in evolution. In the pathways of fixed carbon allocation, the co-factor-independent phosphoglycerate mutase (iPGAM) plays a crucial role by directing the first CO_2_ fixation product, 3-phosphoglycerate, toward central anabolic glycolytic-derived pathways. This work reveals a distinct evolution of iPGAM within oxygenic photosynthetic organisms. We have identified two specific segments in cyanobacterial iPGAMs that affect the control of iPGAM activity through its specific interactor protein PirC. This understanding of iPGAM has allowed us to engineer cyanobacterial strains with altered carbon fluxes. Since cyanobacteria can directly convert CO_2_ into valuable products, our results demonstrate a novel approach for developing a chassis for biotechnical use.

## INTRODUCTION

Phosphoglycerate mutases (PGAMs) are enzymes present in almost every living organism. They connect the major sugar metabolic routes, the Embden–Meyerhof–Parnas pathway, the Entner–Doudoroff pathway, the oxidative pentose-phosphate pathway and its reductive pendant, the Calvin–Benson–Bassham cycle, with the reactions of lower glycolysis. At this metabolic linchpin, PGAM converts 3-phosphoglycerate (3-PGA) into 2-phosphoglycerate (2-PGA), allowing carbon flux into lower glycolysis and thereby into many pathways of anabolic metabolism, such as synthesis of fatty acids or amino acids.

There are two types of PGAM enzymes, which are affiliated with the same superfamily of alkaline phosphates (1): first, the 2,3-bisphosphoglycerate (2,3-BPG)-dependent PGAM (dPGAM) and, second, the 2,3-BPG-independent PGAM (iPGAM) (2–4). Other than dPGAM, iPGAM does not need activation by 2,3-BPG. iPGAMs exist in all plants, algae, some invertebrates, and fungi and are widespread in bacteria (2). In these enzymes, the reversible transfer of the phosphate of the glycerate core from the C3 to the C2 position is achieved in a two-step reaction induced by substrate binding. When no substrate is bound, the cleft between the phosphatase and transferase domains of iPGAMs is open, allowing the entrance of 3-PGA into the binding pocket. Substrate binding induces the closing of the open cleft by phosphatase and transferase domain movement, which enables the reaction to occur (3). 3-PGA first phosphorylates the active-site seryl residue through phosphatase sub-domain activity, followed by reorientation of the substrate and re-phosphorylation of the C2 oxygen by phosphotransferase activity (2). Two manganese ions coordinate the reaction, which makes this reaction highly pH-dependent (4). Species of the *Bacillota* (*Firmicutes*) phylum use this effect to regulate iPGAMs, which play a crucial role in endospore formation (5).

In analogy to the *Firmicutes*, iPGAMs possess a unique regulatory role in cyanobacteria, particularly in response to nitrogen starvation. In *Synechocystis* sp. PCC 6803 (now termed *Synechocystis*), two genes are annotated as iPGAMs, *slr1124* and *slr1945*. The product of *slr1124* was previously identified as a phosphoserine phosphatase and has an essential role in serine biosynthesis in cyanobacteria (6). Notably, we could demonstrate that the product of *slr1945* indeed is an iPGAM, and its regulation is crucial in the biosynthesis of carbon storage polymers during the adaptation to nitrogen-limiting periods (7). The acclimation response of non-diazotrophic cyanobacteria to nitrogen limitation occurs in a process termed chlorosis, which has been thoroughly studied in the strains *Synechococcus* PCC 7942 and *Synechocystis* PCC 6803 (8–10). In the early phase of chlorosis, cells undergo a final doubling before cell cycle arrest, and they immediately start degrading their pigments and forming glycogen as carbon storage (11, 12). Some species, including *Synechocystis* PCC 6803, also accumulate polyhydroxybutyrate (PHB) during the chlorosis process from glycogen turnover (9).

The P_II_ signal transduction protein is a critical factor in the adaptation to nitrogen limitation. It acts as a sensor of the intracellular energy via the binding of ADP and ATP and of carbon-nitrogen status via the binding of 2-oxoglutarate (2-OG). P_II_ directly or indirectly interacts with various enzymes and proteins, affecting a plethora of cellular mechanisms (13, 14). 2-OG binds to P_II_ in synergy with ATP and gives rise to a conformation of P_II_ that prevents interactions with many P_II_ target proteins (reviewed in reference 15). One example of direct interaction is N-acetyl-L-glutamate kinase (NAGK), serving as a model to study P_II_–enzyme interactions (15, 16). More recently, a novel P_II_ interactor, PirA (P_II_-interacting regulator of arginine synthesis), was discovered, whose interaction with P_II_ additionally regulates the NAGK indirectly (17). Activation of the global nitrogen control transcription factor NtcA is indirectly under P_II_ control via the NtcA co-activator PipX (P_II_-interacting protein X). Under low energy or low 2-OG conditions, P_II_ binds and sequesters PipX, whereas this complex dissociates, releasing PipX when 2-OG levels increase due to low nitrogen conditions. Then, PipX co-activates NtcA to stimulate the expression of over 80 genes required for low nitrogen acclimation (18–20).

The newly discovered PirC (Sll0944, P_II_-interacting regulator of carbon metabolism) is like PipX and PirA, a small protein with no enzymatic activity. Still, it modulates the activity of a particular interaction partner, in this case, iPGAM. During nitrogen-supplemented vegetative growth, when 2-OG levels are relatively low, P_II_ forms a complex with PirC, thereby preventing iPGAM inhibition. Upon nitrogen limitation, 2-OG levels increase, and PirC dissociates from the P_II_ complex, which can then inhibit iPGAM. This blocking of the iPGAM stimulates glycogen formation and reduces carbon flow into lower glycolysis, from where many anabolic pathways, including the glutamine synthetase (GS) - glutamate synthase (GOGAT) cycle, are derived (7).

Unlike the regulation of iPGAM in *Bacillota*, the iPGAM in cyanobacteria is regulated via protein–protein interaction with PirC. This unique type of iPGAM regulation in cyanobacteria implies specific structural features of cyanobacterial iPGAMs. Within the iPGAM of cyanobacteria, we identified an internal loop structure and an extended C-terminus (CT). This study aimed to clarify the role of these sub-structures in cyanobacterial iPGAM and their involvement in the PirC interaction by using biochemical and physiological experiments.

## RESULTS

### *In silico* analysis reveals unique sub-domains in cyanobacterial iPGAM

**Fig 1:**
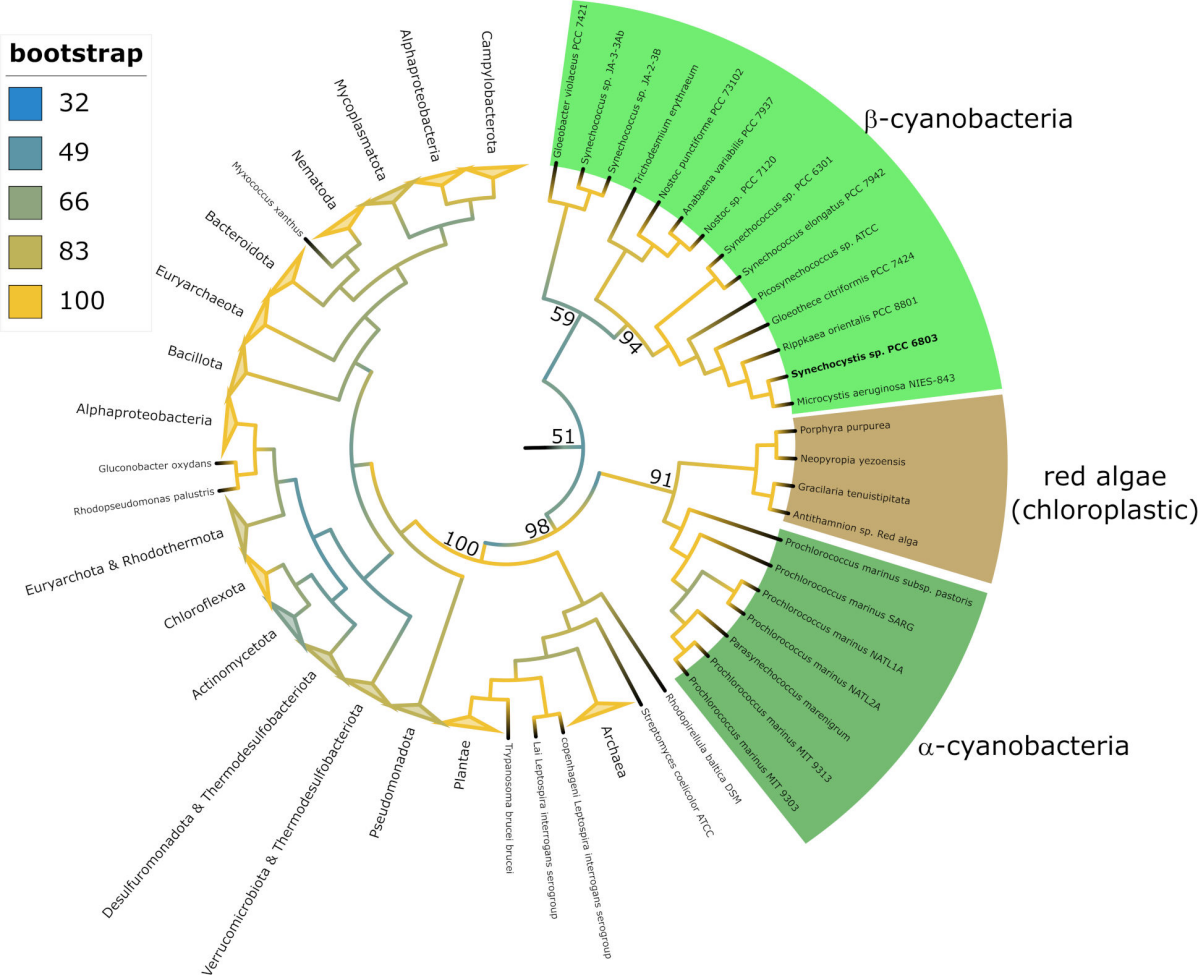
Phylogenetic tree computed with a multiple sequence alignment (MSA) of 338 different iPGAMS (reviewed, according to UniProt). MSA—progressive pairwise alignment using Blosum64 scoring matrix. Tree analysis—maximum likelihood with ultrafast bootstrap analysis (1,000 alignments) computed with IQ-Tree using a general matrix for proteins with a FreeRate heterogeneity +9 (21).

To find out if cyanobacterial iPGAMs differ from other species, we performed a multiple sequence alignment with a simultaneous phylogenetic analysis of 338 different iPGAMs (reviewed according to https://www.uniprot.org) using Matlab and IQ-Tree (21). The tree illustrates the monophyletic evolution of cyanobacterial and red algae iPGAM, distinguishing them from all other tested iPGAMs of bacterial species, plants, and the rare metazoan iPGAMs (bootstrap 98). Furthermore, the tree shows a divergence of iPGAMs from α-cyanobacteria and β-cyanobacteria but with moderate confidence (bootstrap 50). Intriguingly, the chloroplastic iPGAMs of red algae share a common ancestry with the enzymes of cyanobacteria, indicating their origin from the cyanobacterial endosymbiont (bootstrap 91). In contrast, the iPGAM in green plants (kingdom Plantae) is distinct from the cyanobacterial and red algae iPGAMs, indicating that the iPGAM of the endosymbiont was replaced during green plant evolution (Fig. 1).

The multiple sequence alignment revealed two exclusive sequence segments in cyanobacterial iPGAMs: an inner segment of 17 amino acids near the end and an extended CT (Fig. S1). Accordingly, the sequence conservation of the two segments was tested based on multiple sequence alignment of 644 cyanobacterial iPGAMs (Fig. S1B and S1C). The inner segment comprises 17 highly conserved amino acids, which almost always start with a triplet of glutamate, glycine, and glutamate (EGE). The lysine residue at position 6 of the loop is also highly conserved, only replaced by arginine or glutamine and followed by hydrophobic amino acids at positions 14 and 16. There is a high probability of a hydrophobic amino acid, followed by arginine in most cases. By contrast, the CT segment, also unique to cyanobacteria, exhibits poor sequence conservation except for arginine and proline at this segment’s seventh and ninth position, respectively.

**Fig 2:**
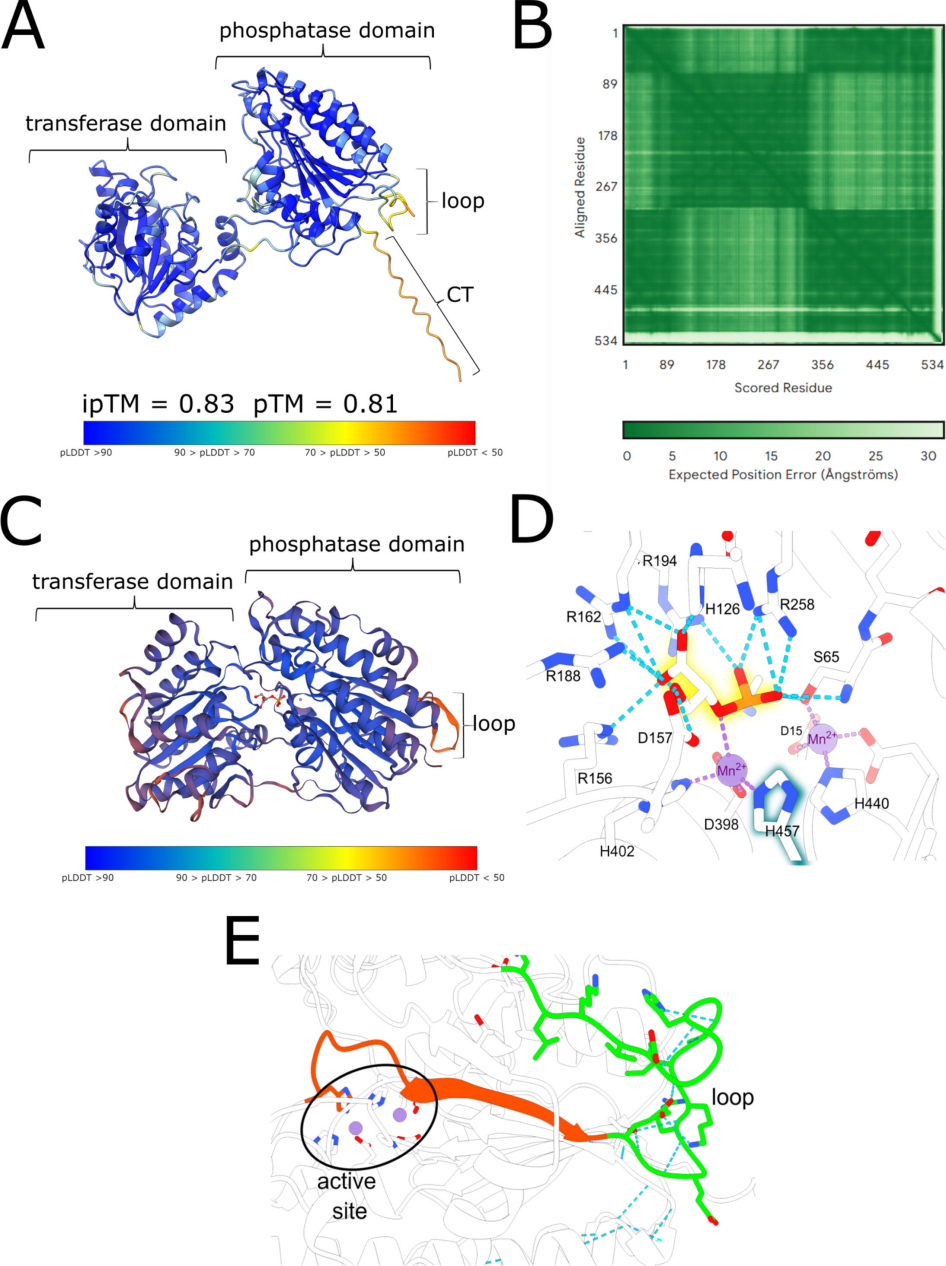
Structure of iPGAM of *Synechocystis* (Slr1945). (A) AlphaFold prediction of the Slr1945. Prediction confidence is shown by the interface predicted template modeling (ipTM) and pTM values and by colorization by the calculated predicted local distance difference test (pLDDT) with the color key below. The AlphaFold server provided by Google DeepMind, which uses the AlphaFold 3 algorithm, was used. (B) Predicted aligned error diagram according to the AlphaFold prediction. (C) SWISS-MODEL prediction of the Slr1945 (PDB template: 1o98, iPGAM of *Geobacillus stearothermophilus*). Prediction confidence is shown by the ipTM and pTM values and by colorization by the calculated pLDDT with the color key below. Exclusive domains and sub-domains are labeled with a bracket. (D) Contributing residues in the catalytic center of Slr1945 according to the SWISS-MODEL prediction. The numbers are based on the position in the *Synechocystis* sequence, shown in Fig. S1D. (E) Connection of the loop to the catalytic center over the β-strand.

For further analysis, the *Synechocystis* iPGAM (Slr1945) structure was predicted using the SWISS-MODEL and AlphaFold server (Google DeepMind) to get more information on Slr1945 and the segments (Fig. 2). The AlphaFold server uses the AlphaFold 3 algorithm. Due to the lack of structural prediction of substrate binding by AlphaFold, the SWISS-MODEL, which shows the bonded substrates, was also used.

The structure predictions of the iPGAM of *Synechocystis* showed the typical division in the phosphatase and transferase sub-domains of iPGAMs. Whereas the AlphaFold prediction shows the open structure without substrate (AlphaFold prediction, Fig. 2A), the SWISS-MODEL in the presence of substrate reveals the closed structure with the bonded substrate (SWISS-MODEL, Fig. 2C) in perfect agreement with the proposed reaction mechanisms. Both predictions revealed the cyanobacterial-specific loop structure of the inner segment. The extended CT was only revealed by Alphafold, predicting a random coil of the CT segment with local proximity to the loop (Fig. 2A and B). The Alphafold prediction was predicted with high accuracy. Most areas have a predicted local distance difference test (pLDDT, a measure for the local confidence of the prediction) value above 70 and close to 90. Additionally, the predicted template modeling (pTM) score of 0.81 and the interface pTM (ipTM) of 0.83 and the predicted aligned error (PAE) graph (Fig. 2B) indicate high confidence in the Alphafold prediction (pTM = measure of the confidence of the whole prediction; ipTM = measure of the confidence of the individual structures within the structure, PAE = graphical presentation of estimated errors between two predicted residues). Only the areas of the exclusive cyanobacterial sub-structures show lower confidence (light areas). Based on SWISS-MODEL prediction, the amino acid residues contributing to the active site of the *Synechocystis* iPGAM could be elucidated (Fig. 2C). It appears that the loop segment is directly connected to the catalytic center, especially to histidine 457 (H457), by 10-amino-acid-long β-strand (Fig. 2D). A closer view into the loop structure revealed direct interaction between the two highly conserved amino acids E468 (glutamine at position 1 according to the segment position) and K473 (lysine at position 6) (Fig. S2).

### Mass photometry indicates the involvement of three PirC monomers in the iPGAM interaction

Recombinant strep-tagged iPGAM and PirC proteins were produced in *Escherichia coli* and purified by Strep-Tactin Superflow affinity chromatography. The purified proteins were analyzed via mass photometry to assess the oligomeric structure of iPGAM, PirC, and the iPGAM–PirC complex. The individual protein measurements used iPGAM concentrations of 10 nM. In preliminary experiments, we tested the optimal ratios for the complex measurement. The iPGAM–PirC ratio of 1:3 showed the most accurate results according to the low appearance of additional peaks (monomers of iPGAM and higher oligomers of PirC) and significant protein counts.

**Fig 3:**
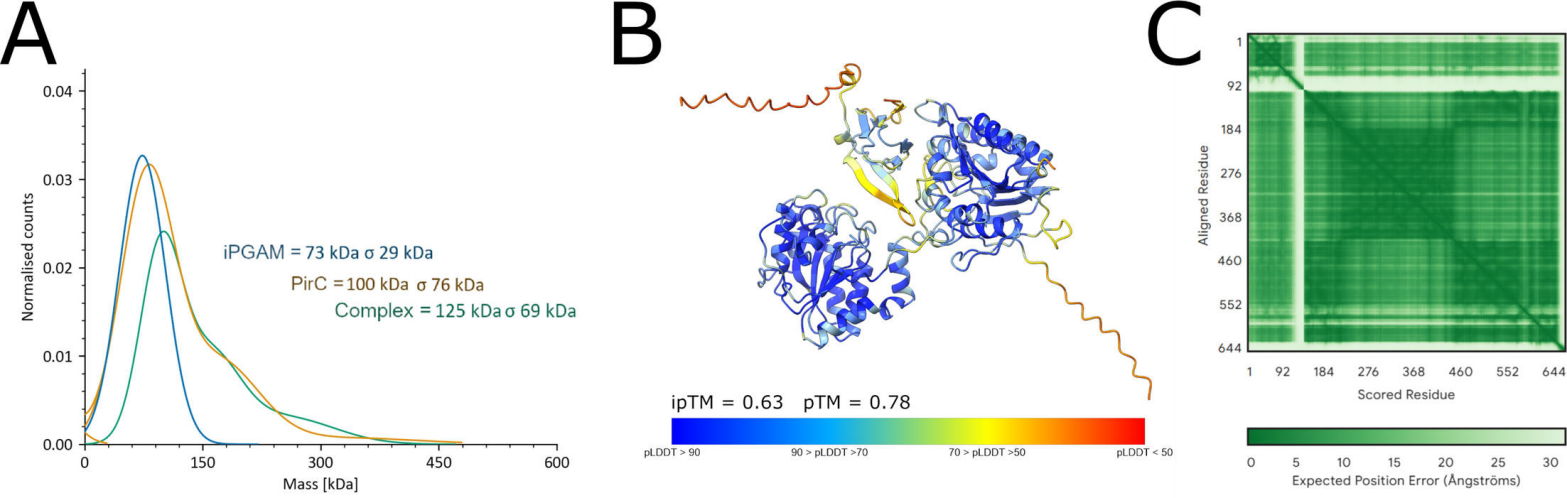
Mass photometry of strep-iPGAM, strep-PirC, and their complex (A), and structure of the iPGAM–PirC complex (B) and the corresponding PAE graph (C). (A) Representative graph of one individual measurement. Triplicates are shown in Fig. S2. (B) Complex of iPGAM and PirC in a ratio of 1:1. Colorized in pLDDT and depicted prediction confidence scores ipTM and pTM. (C) PAE graph based on the complex prediction.

Mass photometry determined monomeric iPGAM particles with masses between 73 and 88 kDa compared to a calculated size of iPGAM of 60 kDa. For purified recombinant PirC, mass photometry determined a maximum of particles between 100 and 114 kDa, fitting to a hexameric structure of the PirC protein (strep-PirC = 14,723.35 Da). Furthermore, a shoulder of around 180 to 210 kDa indicates a higher oligomerized species of PirC. Analyzing the PirC–iPGAM complex resulted in a maximum peak of 125 kDa (replicate 2 = 134 kDa, replicate 3 = 137 kDa). Again, a shoulder appeared at the same position as in the PirC graph. With a size of 125 kDa, the peak is about 55 kDa–65 kDa larger than that of iPGAM alone, which fits the size of three PirC monomers. This suggests the complex could consist of a monomeric iPGAM to which three PirC subunits are bound. According to the results, the structure of the complex was predicted using AlphaFold. However, AlphaFold could not predict such a complex with acceptable confidence (Fig. S4). By contrast, the prediction of a complex only with one PirC bound gave trustable confidence (ipTM of 0.63 and pTM of 0.78) (Fig. 3).

The Alphafold prediction revealed a PirC protomer located in the binding cleft of the substrate between the phosphatase and transferase domains of iPGAM. There, the PirC protomer covers the catalytic center of the phosphatase domain. The confidence of the predicted C-terminus of PirC is low, as indicated by a low pLDDT (Fig. 3A, orange coloring) and a high PAE (Fig. 3B, light areas in the graph)

### Sub-structure-free variants alter the binding of PirC and inhibitory characteristics

**Fig 4:**
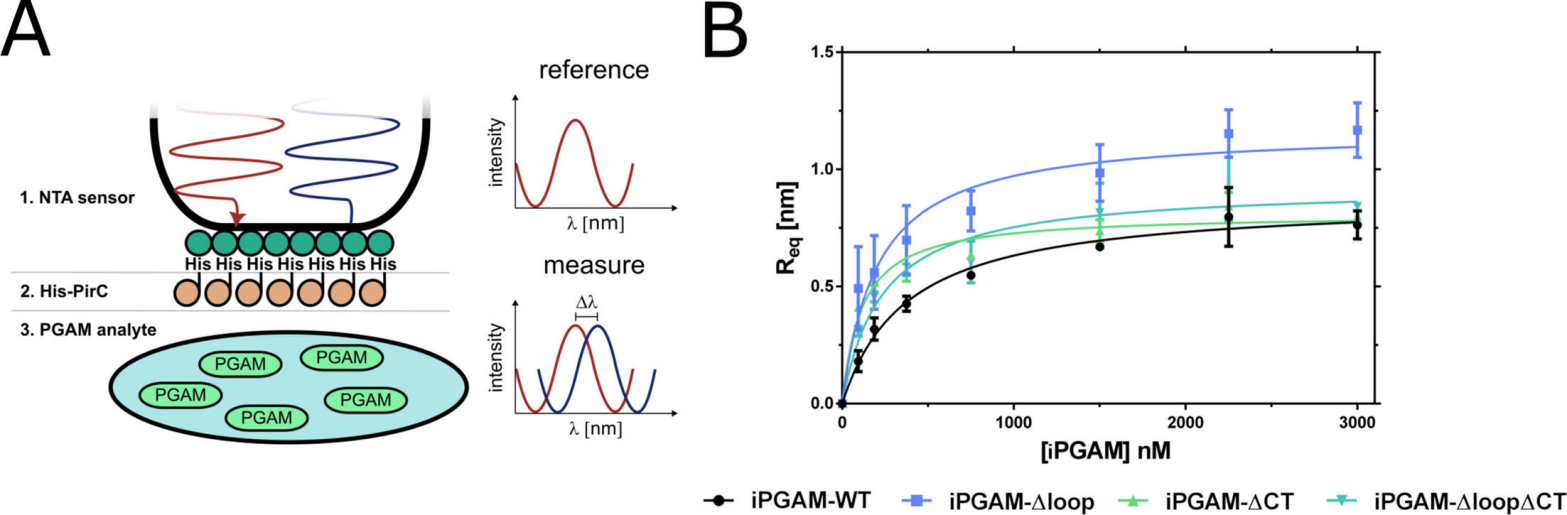
Binding of PirC and iPGAM variants. (A) Principle of BLI measurement with His-PirC and iPGAM variants. In BLI, binding ligands (His-PirC) and analytes (strep-PGAM) at the sensor tips alter the surface’s reflection properties, resulting in a wavelength phase shift. This shift is proportional to the bound molecule. It allows real-time detection of binding, from which the kinetic constants can be calculated. (B) Binding kinetics of iPGAM-WT (wild type), iPGAM-Δloop, iPGAM-ΔCT, and iPGAM-ΔloopΔCT. Each point represents a mean of technical triplicates. The error bars represent the standard deviation.

The exclusive co-occurrence of PirC with the loop and CT segments in cyanobacterial iPGAM implied functional relations. To further investigate the functional significance of these structures, three variants of the *Synechocystis* iPGAM, each with an N-terminal strep-tag, were constructed. First was variant iPGAM-Δloop, where the entire loop was replaced by a five amino acid sequence (TKKGI) present at homologous localization in *Geobacillus stearothermophilus* iPGAM. Second, the CT segment was deleted, creating variant iPGAM-ΔCT, and third, a combination of both alterations, was iPGAM-ΔloopΔCT. In a preliminary experiment, we analyzed the interaction of the various iPGAM variants with PirC by pull-down analysis of Strep-Tactin with immobilized strep-iPGAMs as bait and His-tagged PirC as analyte. Surprisingly, all three variants retained their interaction capacity with iPGAM (Fig. S5). To gain more insight into PirC binding, we performed biolayer interferometry (BLI) measurements using an Octet K2 system. The His-tagged version of PirC was immobilized on the surface of a Ni^2+^-NTA sensor. The different strep-iPGAM variants were then used as analytes in various concentrations for binding kinetics (Fig. 4, Table 1).

**TABLE 1:** Binding kinetic parameters of the different iPGAM variants

Parameter	iPGAM-WT	iPGAM-Δloop	iPGAM-ΔCT	iPGAM-ΔloopΔCT
*R*_max_ [nm]	0.872 ± 0.033	1.170 ± 0.063	0.810 ± 0.025	0.9291 ± 0.047
*K*_*D*_ [nM]	378.4 ± 50.3	203.2 ± 45.0	113.6 ± 18.3	233.4 ± 45.3

The BLI experiments revealed significant differences between the variants. Deleting the loop, CT segment, or both caused an increased affinity for PirC. The *K*_*D*_ was reduced by half in the iPGAM-Δloop and iPGAM-ΔloopΔCT compared to the wild-type (WT). The iPGAM-ΔCT had an even four-times lower *K*_*D*_. The iPGAM-Δloop also had a 35% higher binding maximum than the WT.

**Fig 5:**
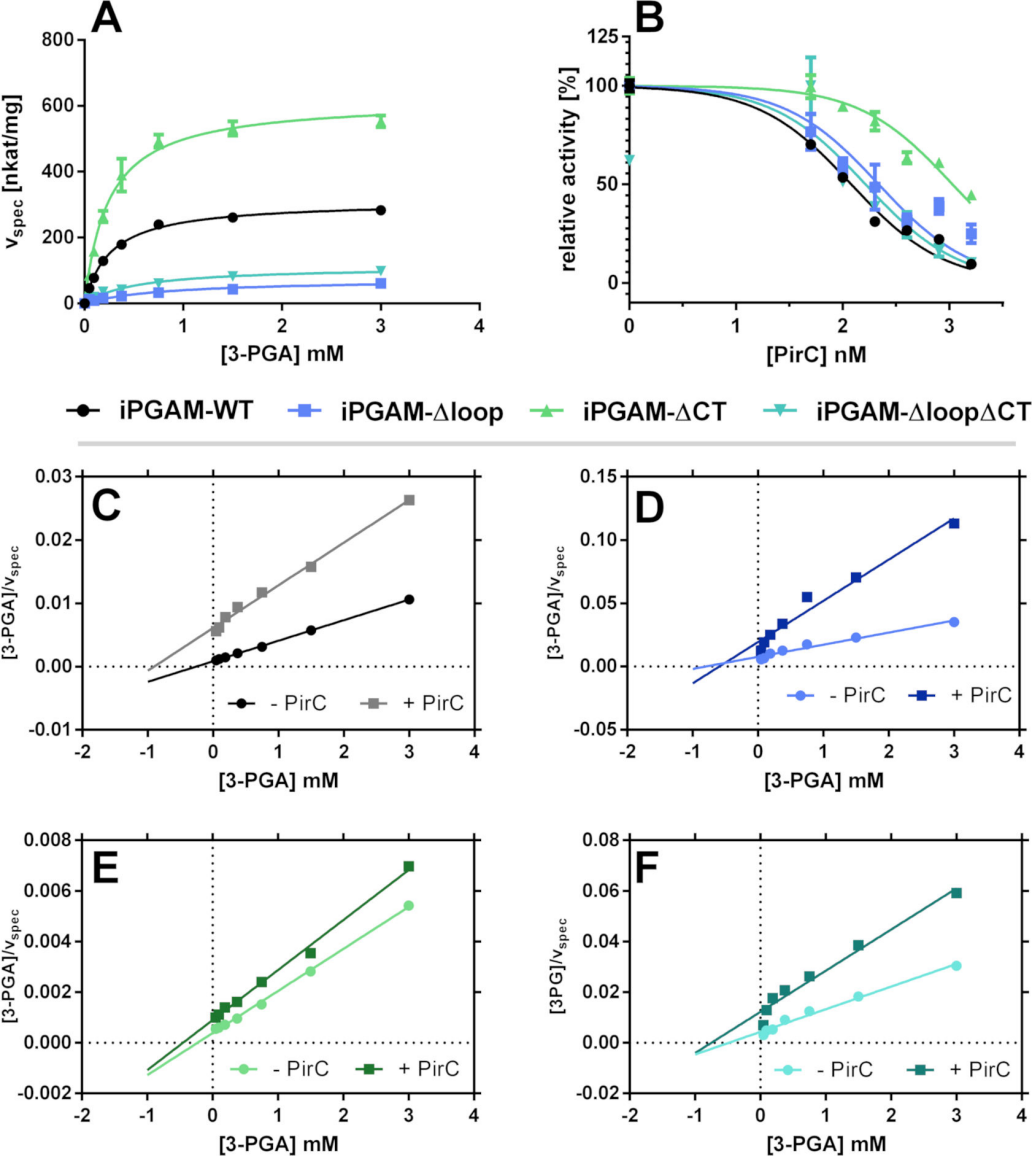
Activity of iPGAM variants of *Synechocystis* sp. PCC 6803 and the effect of PirC on activity. (A) Michaelis-Menten kinetics of iPGAM variants without PirC. (B) Dose-response curve of PirC on the iPGAM variants at 0.75 mM 3-PGA. Each point represents the mean of three independent measured triplicates. The error bar depicts the standard deviation of the triplicate. (C) HW kinetic transformation of WT with and without PirC (400 nM). (D) HW kinetic transformation of Δloop with and without PirC (400 nM). (E) HW kinetic transformation of ΔCT with and without PirC (400 nM). (F) HW kinetic transformation of ΔloopΔCT with and without PirC (400 nM). Each point represents the transformed HW value of the mean of the triplicates. HW transformation → *x*-axis = [substrate concentration], *y*-axis = [substrate]/v.

The BLI experiment proved that the deletion of the loop and CT structures positively affected the binding of PirC. To reveal any effects of these modifications on enzyme activity, coupled enzymatic assays were carried out to determine the catalytic properties of the iPGAM variants and the inhibitory effects exerted by PirC. First, the effect of the co-factor Mn^2+^ was tested to find the optimal manganese concentrations for each iPGAM variant (Fig. S6). The iPGAM-Δloop and the iPGAM-ΔloopΔCT required 20 times more manganese to achieve maximum activity. Therefore, a concentration of 50 µM MnCl_2_ was used in further experiments to achieve the maximum activity with all variants. First, the variants were tested in the absence of PirC (Fig. 5A). Next, the experiments were repeated in the presence of 50 nM, 100 nM, 200 nM, 400 nM, 800 nM, or 1,600 nM PirC to gain information on the inhibition mechanism (Fig. 5 and Table 2). In Fig. 5C through F and Table 2, only the results in the presence or absence of 400 nM PirC are shown for clarity (the full data set is shown in Fig. S7 and S8; kinetic parameters of all concentrations are shown in Table S1). In addition, a dose-response curve of the inhibitory effect of PirC on each iPGAM variant was plotted to calculate the half maximal inhibitory concentration (IC_50_) of PirC, by taking the value at 0.75 mM 3-PGA for each iPGAM assay at the various PirC concentrations (Fig. 5B; Table 3). Moreover, the coupling enzymes were tested with 2-PGA as a substrate to ensure that PirC does not affect the coupling reactions (Fig. S7E). To analyze the inhibition of PirC on the different variants, the Michaelis-Menten (MM) kinetics were transformed to Hanes-Woolf kinetics (HW). The HW transformation of MM kinetics can show the strength and type of inhibition. The steeper the inhibition lines compared to the non-inhibited line, the more potent the inhibition. The convergence of the two lines without crossing before the *y*-axis indicates a non-competitive mode of inhibition (Fig. 5). We observed this for iPGAM-WT (Fig. 5C). Additionally, the increased *K*_*m*_ of the iPGAM-WT–PirC complex indicates competitive inhibiting properties, whereas the decreased *v*_max_ again showed non-competitive inhibition. This implies a type of mixed inhibition by PirC (Table 2).

**TABLE 2:** Kinetic parameters of iPGAM-WT, -Δloop, -ΔCT, and -ΔloopΔCT^*a*^

Sample iPGAM	PirC	*K*_*m*_ (mM)	*v*_max_ (nkat · mg^−1^)	*k*_cat_ (s^−1^)	*k*_cat_ · *K*_*m*_^−1^ (s^−1^· M^−1^)
WT	−	0.265 ± 0.013	310.6 ± 4.5	18.6 ± 0.3	70,241.7 ± 3,588.3
	+	1.018 ± 0.070	154.4 ± 4.5	9.3 ± 0.3	9,096.3 ± 681.0
Δloop	−	1.087 ± 0.141	114.3 ± 6.4	6.7 ± 0.4	6,189.5 ± 873.9
	+	0.750 ± 0.132	32.1 ± 2.2	1.9 ± 0.1	2,522.3 ± 475.9
ΔCT	−	0.242 ± 0.019	617.9 ± 13.7	35.9 ± 0.8	148,468.5 ± 11,979.3
	+	0.482 ± 0.056	521.4 ± 20.7	30.3 ± 1.2	62,813.9 ± 7,711.8
ΔloopΔCT	−	0.547 ± 0.059	112.8 ± 4.3	6.4 ± 0.2	11,760.0 ± 1,355.4
	+	0.961 ± 0.182	65.7 ± 5.2	3.7 ± 0.3	3,900.7 ± 802.5

^*a*^ Values represent the mean of triplicates with (+) and without (−) the addition of 400 nM PirC . The error range of *K*_*m*_, *v*_max_, and *k*_cat_ represents the standard error calculated by GraphPad Prism. The error of *k*_cat_ · *K*_*m*_^-1^ was calculated using error propagation.

**TABLE 3:** IC_50_ of PirC with the different iPGAM variants^*a*^

Sample	IC_50_ (nM)
iPGAM-WT	120.1 ± 1.06
iPGAM-Δloop	217.0 ± 1.14
iPGAM-ΔCT	1,069 ± 1.07
iPGAM-ΔloopΔCT	163.5 ± 1.25

^*a*^ The IC_50_ was detected at 0.75 mM 3-PGA. Values represent the mean of triplicate. The error range represents the standard error calculated by GraphPad Prism.

Compared to iPGAM-WT, the iPGAM-Δloop variant had three times lower activity but was still inhibited by PirC. Interestingly, inhibition was less efficient, with a twofold increase in IC_50_ for PirC compared to inhibiting the iPGAM-WT (IC_50, iPGAM-WT_ = ~120 nM, IC_50, iPGAM-Δloop_ = ~220 nM). More striking differences were observed for the iPGAM-ΔCT variant. First, the catalytic efficiency (CE) of iPGAM-ΔCT was severely enhanced with a CE value of 150,000 s^−1^· M^−1^ compared to ~70,000 s^−1^· M^−1^ for WT. Furthermore, the inhibitory effect of PirC on iPGAM-ΔCT was almost completely lost, as shown by the small difference in slope between the non-inhibited and inhibited states (Fig. 5E). The inhibited iPGAM-ΔCT still had a CE of 63,000 s^−1^ · M^−1^, which is close to the activity of non-inhibited iPGAM-WT (Table 2). The dose-response curves of PirC and the calculated IC_50_ demonstrated a 10-fold decreased efficiency of PirC to inhibit iPGAM-ΔCT compared to iPGAM-WT (IC_50, iPGAM-WT_ = ~120 nM, IC_50, iPGAM-ΔCT_ = ~1070 nM, see Table 3). The iPGAM-ΔloopΔCT variant combined properties of the two single mutations: deleting the C-terminus in the iPGAM-Δloop variant increased its catalytic efficiency and partially decreased the inhibitory effect of PirC compared to the Δloop variant (Table 2).

### Sub-domain-free variants influence the physiology during chlorosis

To investigate the effect of the above-described alterations of the iPGAM variants on the physiology of *Synechocystis*, mutant strains were generated by homologous recombination of native iPGAM with the iPGAM variants followed by spectinomycin (Δloop) or chloramphenicol (ΔCT and ΔloopΔCT) as selection markers. As controls, the WT was used as a reference for the standard interaction between iPGAM and PirC and a PirC deletion mutant (ΔPirC) for native iPGAM without PirC regulation.

**Fig 6:**
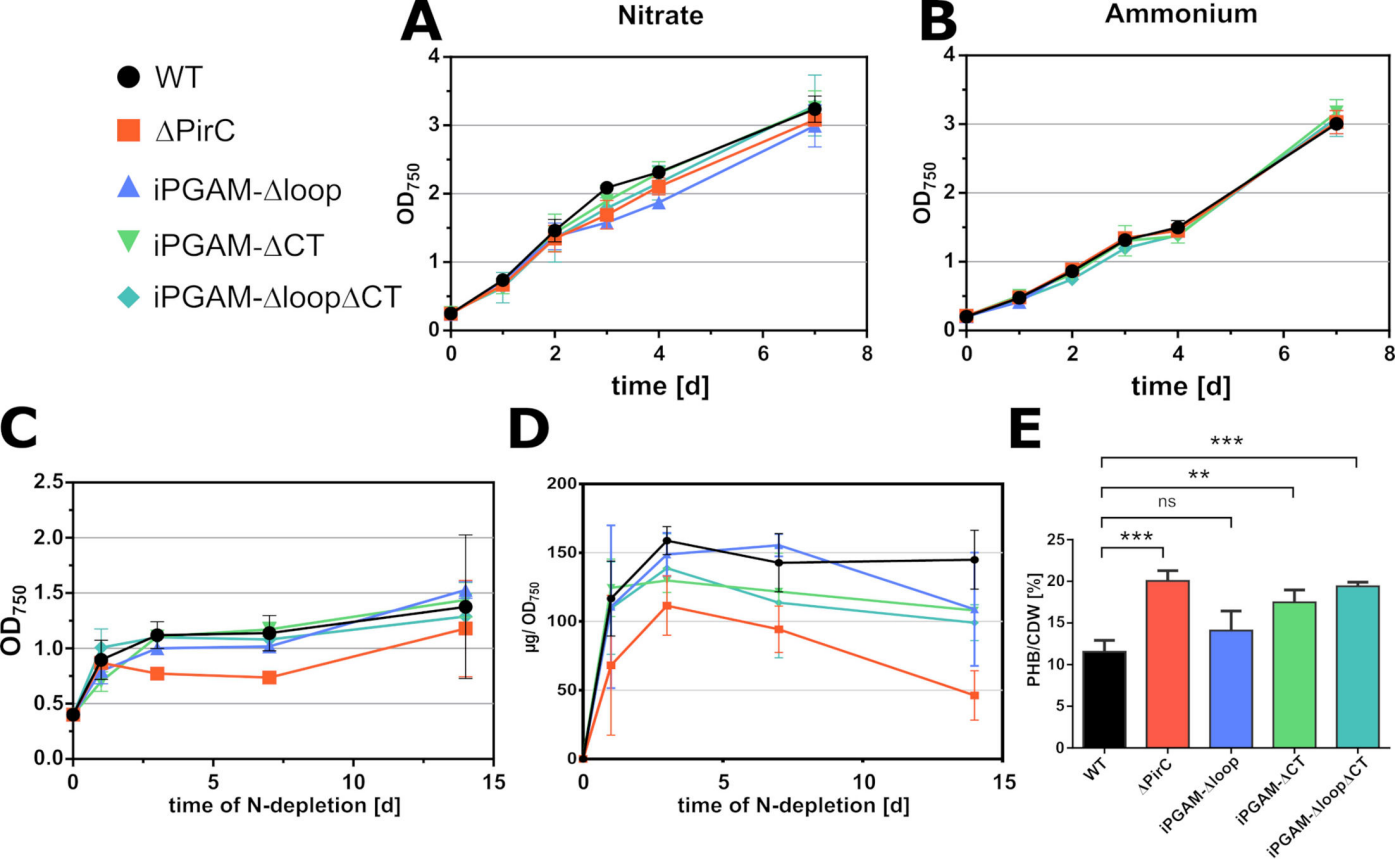
Effect of iPGAM-Δloop, -ΔCT, and -ΔloopΔCT on the physiology of *Synechocystis* sp. PCC 6803. (A) Growth curves of iPGAM variants with 17 mM NaNO_3_. (B) Growth curves of iPGAM variants with 5 mM NH_4_Cl. (C) OD_750_ of iPGAM variants during nitrogen depletion. (D) Glycogen content in iPGAM variant strains during nitrogen depletion. Each point represents the mean, and the error bars represent the standard deviation of independent triplicates. (E) PHB content after 14 days of nitrogen depletion in the iPGAM variants. Each bar and error bar represent the mean of biological triplicates. Statistical analysis was performed using a one-way analysis of variance. Each column was compared to the WT control. Dunnett’s multiple comparisons test was used (*P* < 0.05).

Since the PirC–iPGAM interaction affects physiology during nitrogen limitation, we tested the effect in nitrogen deprivation experiments, in which we analyzed the OD_750_, the glycogen amount, and the PHB levels after 14 days. As control experiments, we also tested the effect of nitrogen-rich conditions with nitrate or ammonia (Fig. 6).

All *Synechocystis* variants showed similar growth in the standard BG_11_ medium and reached a comparable maximal optical density (OD_750_) after 7 days of cultivation. Also, when ammonium was used as a nitrogen source, no differences between the strains showed up. However, upon nitrogen step-down, phenotypic differences to the wild-type became apparent. Upon nitrogen depletion, the wild-type carries out a final cell division before arresting the cell cycle, which is evidenced by an increase in OD_750_ until reaching a stationary value, which is double the initial OD_750_ (8, 10, 22). The ΔPirC strain, by contrast, was unable to carry out the doubling of OD_750_. In contrast to the ΔPirC strain, the iPGAM variants exhibited the final increase in OD_750_ like the wild-type. The ΔPirC accumulated less glycogen and had twice as high levels of PHB compared to the WT. The iPGAM-Δloop mutant accumulated glycogen at similar levels as the WT but required more time to reach its maximum level. The iPGAM-ΔCT and -ΔloopΔCT strains showed intermediary phenotypes between ΔPirC and WT for glycogen accumulation. A hallmark of the *pirC* mutation was the strongly increased accumulation of PHB. In this respect, the iPGAM-ΔCT and -ΔloopΔCT strains resembled the ΔPirC strain. After 14 days of chlorosis, the PHB level per cell dry weight (CDW) of iPGAM-ΔCT (18.2 ± 0.4 mg · CDW^−1^ ) and ΔloopΔCT (19.4 ± 0.3 mg · CDW^−1^) strains reached PHB levels similar to the high PHB-producer ΔPirC (20.1 ± 1.0 mg · CDW^−1^), whereas those of the iPGAM-Δloop strain were similar to the WT (12.9 ± 0.5 mg · CDW^−1^ compared to 1.5 ± 1.5 mg · CDW^−1^).

## DISCUSSION

This research unveils a unique insight into the pivotal role of the iPGAM in regulating carbon flux in cyanobacteria. Our bioinformatics analysis revealed two structural elements exclusively in cyanobacterial iPGAM, indicating a unique functional connection with PirC. The phylogenetic analysis showed that cyanobacterial iPGAMs diverged early from iPGAMs of other species. Notably, the iPGAMs of red algae show a phylogenetic relation to cyanobacterial iPGAM despite lacking the two characteristic segments. The chloroplasts of red algae have retained several features from the early endosymbiont, which were later lost in the evolution of green plants, such as components of the light-harvesting system. Furthermore, red algae exhibit P_II_ signaling features that resemble cyanobacteria, such as N-acetyl-L-glutamate kinase regulation by P_II_ (23). The phylogenetic tree of cyanobacterial iPGAM corresponds strikingly well with a recent whole-genome phylogeny of cyanobacteria (24), which considers many more genes than the usually used 120 housekeeping genes (25). If this refined phylogenetic tree accurately reflects the evolution of cyanobacteria, it suggests that the characteristic features of iPGAM and their regulation by PirC emerged early in cyanobacterial evolution. Alternatively, the prominent divergence of iPGAM homology between α- and β-cyanobacteria would indicate a different trend in protein evolution in these cyanobacterial groups. In red algae, the evolutionary loss of PirC led to the disappearance of the CT and loop segments, but other features of iPGAM were conserved, maintaining the phylogenetic clustering. In contrast, the evolution toward green plants involved significantly streamlining the P_II_ signaling system along with metabolic rearrangements. The endosymbiont’s iPGAM was replaced by an unrelated enzyme, not associated with P_II_ signaling, along with the translocation of the *glnB* gene to the nucleus and the reorganization of the P_II_ signaling system (such as the acquisition of the glutamine-sensing C-terminal domain).

Detailed kinetic analysis of iPGAM now shows characteristics of both competitive and non-competitive inhibition by PirC. The enzyme has a lowered affinity to the substrate in the presence of PirC, as demonstrated by a lowered *K*_*m*_. Furthermore, the iPGAM also has a reduced maximal activity in the presence of PirC (shown by *v*_max_ and *k*_cat_). The PirC peptide, which AlphaFold predicted to be localized in the phosphatase-transferase interdomain cleft, possibly hinders the cleft closure, which is required to perform the reaction. Thereby, the catalytic reaction is inhibited.

The replacement of the loop reduced the activity of the iPGAM, reaching only one-third of the maximal activity as the iPGAM-WT, and the increased *K*_*m*_ evidence four times decreased substrate affinity. Residue H457 in the catalytic center in *Synechocystis* iPGAM, equivalent to H462 from the *G. stearothermophilus* iPGAM, is connected via a β-strand to the loop. This histidine residue contributes to the binding of manganese (Fig. 2D) (26). In agreement, the iPGAM-Δloop variant has a reduced affinity to Mn^2+^ (*K*_half, WT_(Mn^2+^) = ~1.3 µM; *K*_half, Δloop_(Mn^2+^) = ~10.5 µM), and the enzyme requires much higher Mn^2+^ concentrations to reach its maximal activity. The AlphaFold prediction of the iPGAM structure revealed an H-bond between K473 and E468 within the loop. This interaction possibly stabilizes the β-strand connection to the catalytic center, favoring the manganese binding. The iPGAM of *G. stearothermophilus,* which does not possess this loop, requires manganese concentrations that are 1,000-fold higher than the *K*_half_(Mn^2+^) of *Synechocystis* iPGAM for maximal activity (27). The low activity of the iPGAM-Δloop indicates that the lowered manganese affinity consequently affects the whole reaction. Hence, the H457 residue contributes to the coordination of the bond Mn^2+^, which is essential in the phosphatase reaction of the enzyme. The H-bond K473-E468 within the loop structure keeps the H457 in an optimal position, and Mn^2+^ binds with higher affinity. Furthermore, the Mn^2+^ coordinates the phosphoester bond of 2- or 3-PGA and thus enables the hydrolyzation of the phosphate. In the iPGAM-Δloop variant, the K473-E468 interaction does not exist, and H457 could be in another orientation within the catalytic center, resulting in a lowered *K*_*m*_. Intriguingly, the iPGAM-Δloop variant has a higher affinity for PirC, indicating a conformational change imposed by the loop deletion that facilitates PirC interaction.

In contrast to the iPGAM-Δloop, the iPGAM-ΔCT has a twofold increased *v*_max_ compared to iPGAM-WT, whereas the *K*_*m*_ is not altered. At the same time, the affinity toward PirC is strongly enhanced, but PirC only weakly inhibits the iPGAM reaction: the inhibitory effect of PirC on iPGAM-ΔCT activity is 10-fold lower than on iPGAM-WT (IC_50, PirC_(iPGAM-WT) = ~120 nM, IC_50, PirC_(iPGAM-ΔCT) = ~1,070 nM). This directly implies that the C-terminal flexible extension plays a crucial role in modulating the activity of iPGAM and transmits the inhibitory effect of PirC binding to the catalytic center. Possibly, this C-terminal extension lowers the *v*_max_ of the reaction by interfering with the domain closure. By removing this tail, the enzyme can work at its maximum pace. In agreement, the binding of PirC would place the C-terminal tail in a position where the inhibitory effect is augmented. Without the C-terminal extension, the binding of PirC cannot exert this inhibitory function but facilitates its binding to the iPGAM body. This can be explained by the assumption that the binding of the CT extension by PirC is thermodynamically unfavorable. Residual inhibition of the iPGAM-ΔCT variant requires 10-fold higher concentrations of PirC, although the affinity of these partners has increased, which appears counterintuitive. It suggests that additional binding of PirC protomers to low-affinity binding sites of iPGAM-ΔCT is required to achieve inhibition. These additional binding events may impair the catalytically driven domain closure of iPGAM. Additional binding sites also agree with the experimentally observed size of the iPGAM–PirC complex, suggesting that more than one protomer of PirC can bind to iPGAM.

Concerning the iPGAM-Δloop-ΔCT variant, it shows a similar increased *K*_*m*_ for Mn^2+^ as the single iPGAM-Δloop, which agrees with the obvious role of the loop segment in high Mn^2+^ affinity. Surprisingly, however, the combined removal of both the loop and the CT segments gave rise to compensatory effects concerning its catalytic properties, as the catalytic efficiency is in between that of iPGAM-Δloop and iPGAM-WT variants. The inhibition of iPGAM-Δloop-ΔCT variant activity by PirC affects the *K*_*m*_ in a similar way as iPGAM-ΔCT. Overall, the binding of PirC to different sites of iPGAM, sites near the unique loop structure, and the CT segment is likely the structural basis of the observed mixed-type inhibition. Although we showed that the substructures play an important role in iPGAM inhibition, PirC still inhibits all enzyme variants to some extent. This fits with the prediction of the PirC protomer within the binding cleft of iPGAM, which is independent of the sub-structures. To elucidate the details of the mechanism and how the loop and the C-terminus influence the inhibitory effect of PirC on iPGAM activity, it is necessary to solve the actual structure of the PirC–iPGAM complex.

Under nitrogen-replete conditions, all the iPGAM variant strains grow similarly to the WT. This shows that during nutrient-replete vegetative growth, the low activities of the iPGAM-Δloop and iPGAM-ΔloopΔCT variants are sufficient to maintain metabolic homeostasis. Conversely, the excessive activity of the iPGAM-ΔCT variant during vegetative growth has neither a positive nor a negative effect on *Synechocystis* growth. However, a distinct phenotype of iPGAM variant strains appeared during nitrogen starvation. Unlike the ΔPirC strain, the iPGAM-Δloop, -ΔCT, and -ΔloopΔCT strains could carry out the final doubling of OD_750_ upon shifting to a nitrogen-depleted medium. Furthermore, all iPGAM strains formed similar amounts of glycogen, whereas the ΔPirC strain is strongly affected by glycogen accumulation, in agreement with earlier observations (7). During prolonged chlorosis, the iPGAM-Δloop, -ΔCT, and -ΔloopΔCT strains showed slightly increased glycogen degradation compared to the WT. Previously, we showed (7) that the levels of 3-PGA, a key activator of glycogen formation, in both the WT and the ΔPirC mutant doubled within the first 6 hours after N depletion, indicating that this initial 3-PGA accumulation does not require iPGAM inhibition by PirC. This also explains the initial glycogen increase in ΔPirC. During further nitrogen starvation, expression of PirC is strongly induced (28). This leads to a pronounced inhibition of iPGAM and, consequently, a further increase of 3-PGA levels in the wild-type, which is no longer observed in the ΔPirC. Consequently, glycogen synthesis continues in the WT to reach its maximal levels after approximately 2 days, whereas glycogen synthesis prematurely slows down in ΔPirC.

During prolonged nitrogen starvation, glycogen is slowly converted into PHB (9). In the ΔPirC strain, lack of iPGAM inhibition during chlorosis leads to accelerated carbon flow into lower glycolysis, finally resulting in PHB formation. Both strains expressing the CT-truncated iPGAM (iPGAM-ΔCT and iPGAM-ΔloopΔCT) show almost the same amount of PHB accumulation after 14 days of nitrogen starvation as the ΔPirC. This can very likely be attributed to the reduced inhibition of these variants by PirC, whereas the iPGAM-Δloop strain shows similar levels to the WT, in agreement with the low activity of the iPGAM-Δloop–PirC complex.

Although the iPGAM-ΔCT-expressing strains produce similar amounts of PHB during prolonged chlorosis as the PirC-deficient strain, they still are able to respond to nitrogen starvation in a similar way to the WT by accumulating glycogen and performing a final cell division, whereas, in the absence of PirC, the cells go immediately into growth arrest. This could indicate additional roles of PirC beyond iPGAM inhibition for the acclimation toward nitrogen starvation. However, concerning the effect on PHB accumulation, inhibition of iPGAM seems to be the most important function. Previously, Koch et al. (29) achieved higher PHB levels with the ΔPirC strain by introducing additional *phaA* (acetyl-CoA acetyltransferase) and *phaB* (acetoacetyl-CoA reductase) genes, whose products catalyze the initial steps of PHB synthesis. A similar approach should also increase the PHB amounts in iPGAM-ΔCT or the iPGAM-ΔloopΔCT. In contrast to ΔPirC, these strains do not have the disadvantage of biomass loss during nitrogen starvation, which seems beneficial for biotechnological applications.

## MATERIALS AND METHODS

Detailed descriptions of the methods are shown in the supplemental methods.

### Multiple alignments and phylogenetic tree calculation of phosphoglycerate mutases

The alignments were done with Matlab and the tree was computed with IQ-Tree (21). The resulting tree was visualized with iTol (30).

### Structure predictions

The structure of the iPGAM was predicted using the SWISS-MODEL workspace and AlphaFold server (31–33). AlphaFold was also used to predict the structure of the complex.

### Molecular cloning and mutagenesis

Gibson assembly (GA) and mutagenesis PCR were used to create the plasmids. The GA was done according to the manufacturer protocol (NEB E2611S/L, E5510S).

According to the manufacturer protocol, iPGAM was mutated with the Q5 Site-Directed Mutagenesis Kit (NEB, E0554).

### Plasmid and strains

The physiological experiments on cyanobacteria were carried out with the unicellular non-diazotrophic *Synechocystis* sp. PCC 6803 wild-type glucose sensitive strain, which is based on the Kazusa strain. All created mutants are also based on the above-described background strain.

Plasmids and all other strains created and used in this study are listed in Table S1 and Table S2.

### Cultivation of cyanobacteria

Growth experiments and precultures of *Synechocystis* were cultivated in BG_11_, and the composition was explained by Mager et al. (34). Standard cultivation was performed at 28°C with continuous shaking at 125 rpm at constant illumination (24 h · d^–1^, ∼50 μE m^–2^· s^–1^). The BG_11_ was adjusted for different experiments, as explained in the supplemental material.

For nitrogen deficiency experiments, precultures of *Synechocystis* at an OD_750_ of 0.6–1 were washed with and resuspended in BG_11_,0 medium, and the nitrogen-free culture was inoculated to an OD_750_ of 0.4.

*Escherichia coli* cultures were grown on lysogenic broth (LB) medium and agar.

### Expression and purification of proteins

*E. coli* Lemo21(DE3) was used to overexpress proteins induced depending on the vector either by 400 mM isopropyl-β-D-thiogalactopyranoside (IPTG) or 200 µg anhydrotetracycline. The His-tagged proteins were purified using HisTrap HP columns (Cytiva, Marlborough, USA) and strep-tagged protein using the Strep-Tactin Superflow columns (IBA Lifescience, Göttingen, Germany) by affinity chromatography.

### Mass photometry using the Refeyn OneMP

A mass photometry experiment was used to study the variants’ oligomerization and the stoichiometry of the iPGAM–PirC complex. For this purpose, the Refeyn OneMP was used. The data were analyzed using the DiscoverMP software.

### BLI using the Octet K2 system

*In vitro* binding studies were done using BLI using the Octet K2 system (Sartorius, Göttingen, Germany) according to the Bio-Protocol (35).

### Phosphoglycerate mutase assay

The iPGAM activity was determined by a coupled enzyme assay as adapted as described previously (7). The release of the product 2-PGA, by iPGAM, is coupled to enolase, pyruvate kinase, and lactate dehydrogenase (LDH). LDH transforms NADH to NAD^+^ by its reaction, and the NADH decrease was measured spectrophotometrically at 340 nm.

### Glycogen measurement

The glycogen content was quantified according to previous studies from 2 mL *Synechocystis* culture samples (36). Samples of 2 mL cell culture were lysed by boiling at 95°C in 30% (wt/wt) KOH. The released glycogen was washed, accumulated, and enzymatically cleaved to glucose and detected via reaction with o-toluidine and measurement of the resulting compound spectrophotometrically at 635 nm.

### PHB quantification

PHB was detected using high-performance liquid chromatography as described previously (7, 29, 37).

## SUPPLEMENTAL MATERIAL

Supplemental material mbio.03378-24-s0001.docx Figures S1 to S8, Tables S1 to S4, and supplemental methods.

ASM does not own the copyrights to Supplemental Material that may be linked to, or accessed through, an article. The authors have granted ASM a non-exclusive, world-wide license to publish the Supplemental Material files. Please contact the corresponding author directly for reuse.
