# Supplementary material for: Structural elements of cyanobacterial co-factor-independent phosphoglycerate mutase that mediate regulation by PirC
Source: mBio. 2025 Apr 3;16(5):e03378-24. doi: 10.1128/mbio.03378-24 (PMC12077085; doi:10.1128/mbio.03378-24)
Supplement: Supplemental material — Figures S1 to S8, Tables S1 to S4, and supplemental methods. [file mbio.03378-24-s0001.docx]

# Supplemental Material

## Supplementary Results


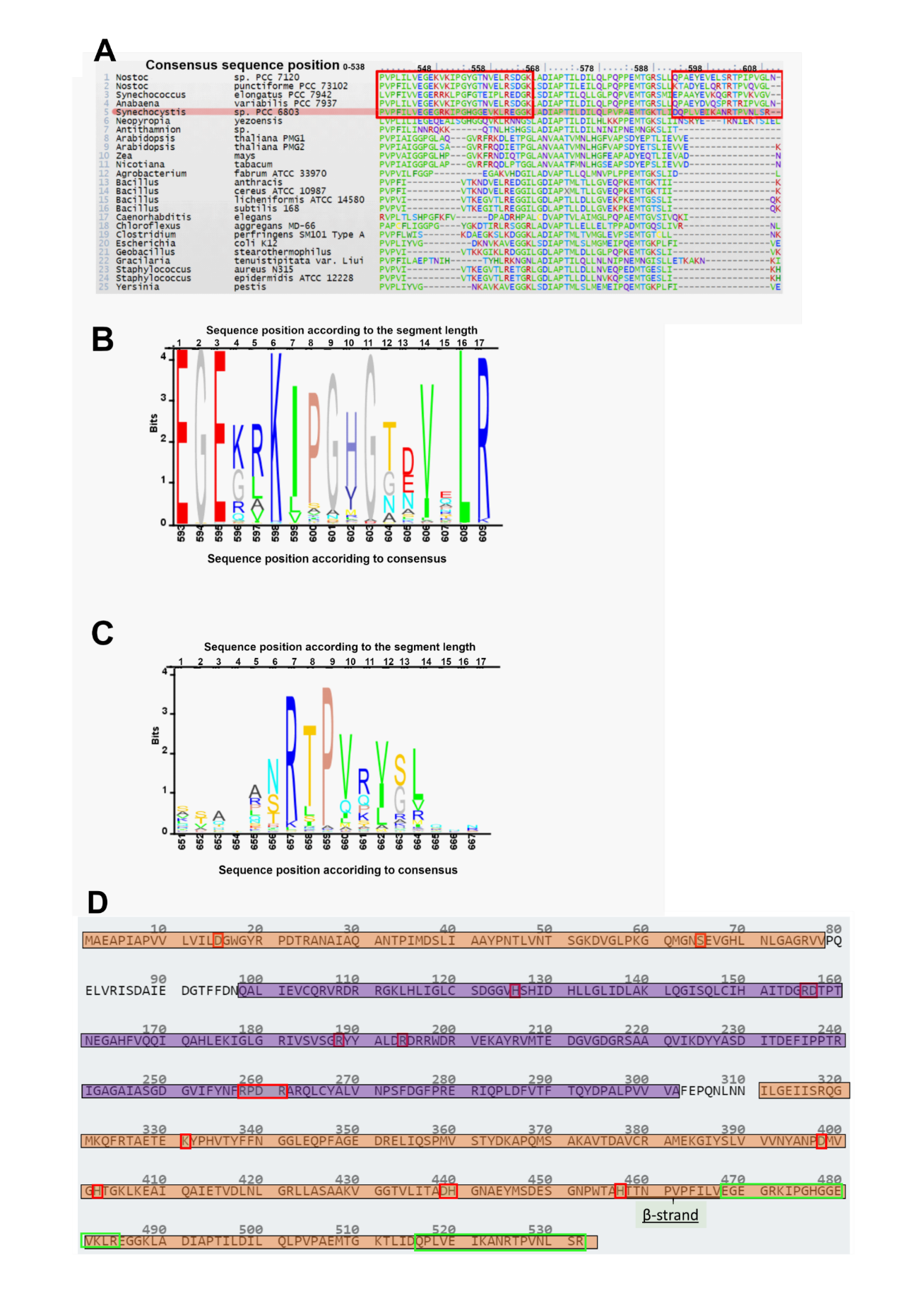


Figure S 1 – Comparison of 338 reviewed iPGAM sequences (uniprot.org) and 644 iPGAM sequences of cyanobacteria revered as active (uniprot.org). (A) Excerpt of an alignment of 338 different iPGAMs. Cyanobacteria have two unique sequences, which are highlighted by a red box. The first is called a loop according to the predicted structure. Second, an extended C-terminus. (B) The sequence logo of the loop sequence was calculated via an alignment of iPGAMs of 644 different cyanobacteria. (C) Sequence Logo of the extended C-Terminus in Cyanobacteria. (D) Sequence of Slr1945 with highlighted Domains and contributing amino acids in the catalytic centre. Orange = phosphatase domain, Purple = transferase domain. Red boxes = catalytical amino acid residues, green boxes = loop and CT segments.


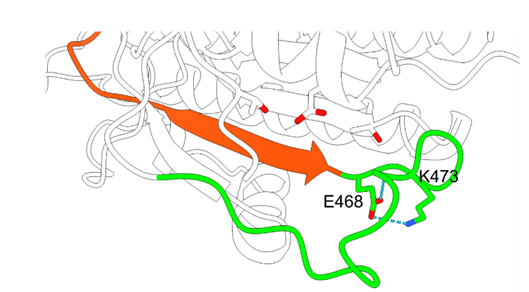


Figure S 2 – Hydrogen bond interactions H-Bond of E468 and K473 within the loop


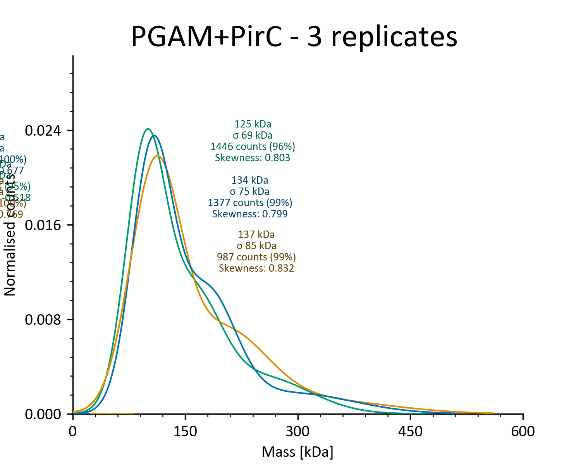

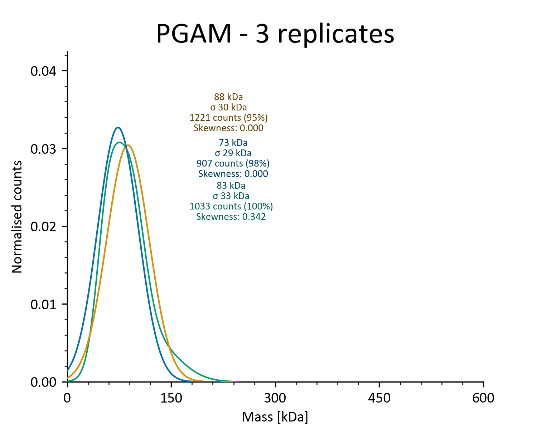

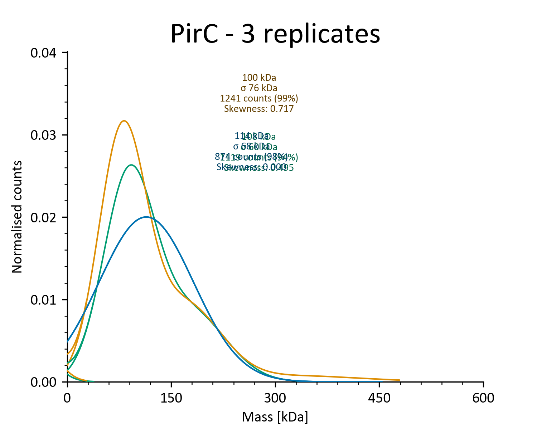


A

B

C

Figure S 3 – Mass Photometire of three independent technical replicates of strep-PGAM-WT (A), strep-PirC (B) and the complex of both (C). Gaussian fitting of the counts and the calculated mass of the major mass of the fit. Each bar represents the normalized count


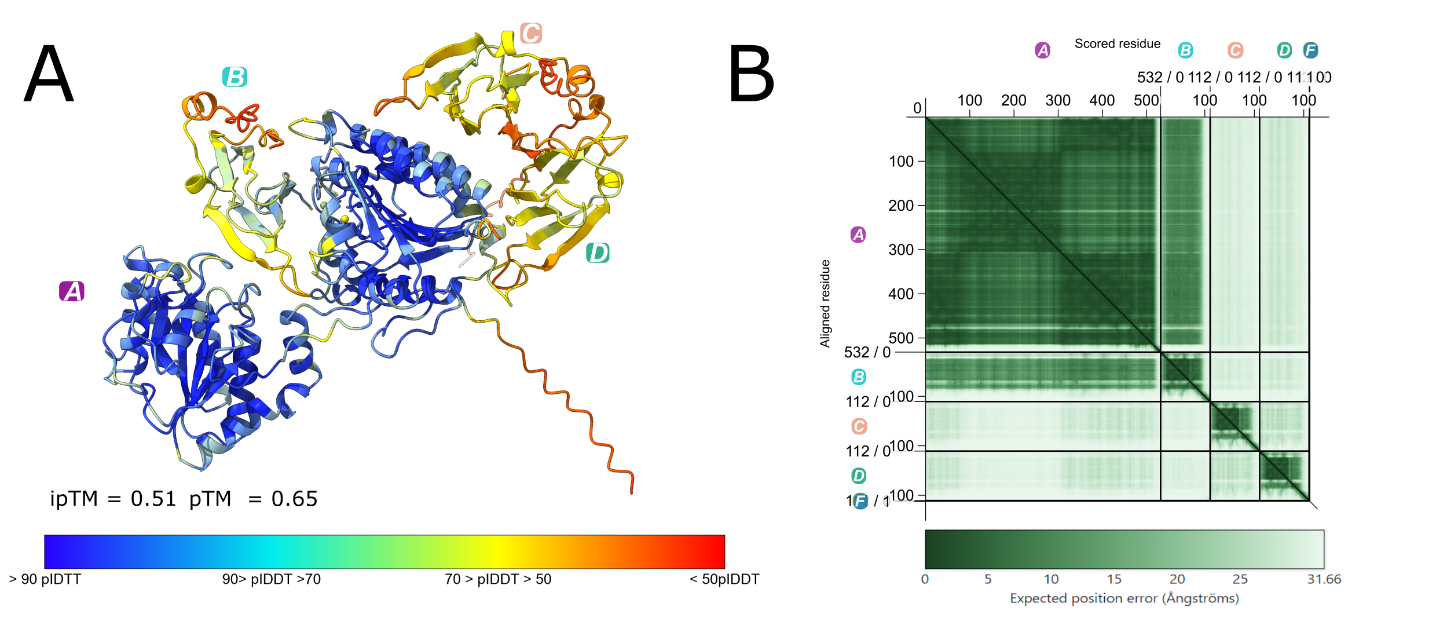


Figure S 4 – Structure of *Synechocytsis* PGAM in complex with three monomers of PirC. (A) AlphaFold-predicted structure of the PGAM-PriC(tri) colourized by the predicted IDDT matching score (pIDDT) of the structure (B) Predicted aligned error (PAE) diagram of the PGAM-PriC(tri) complex


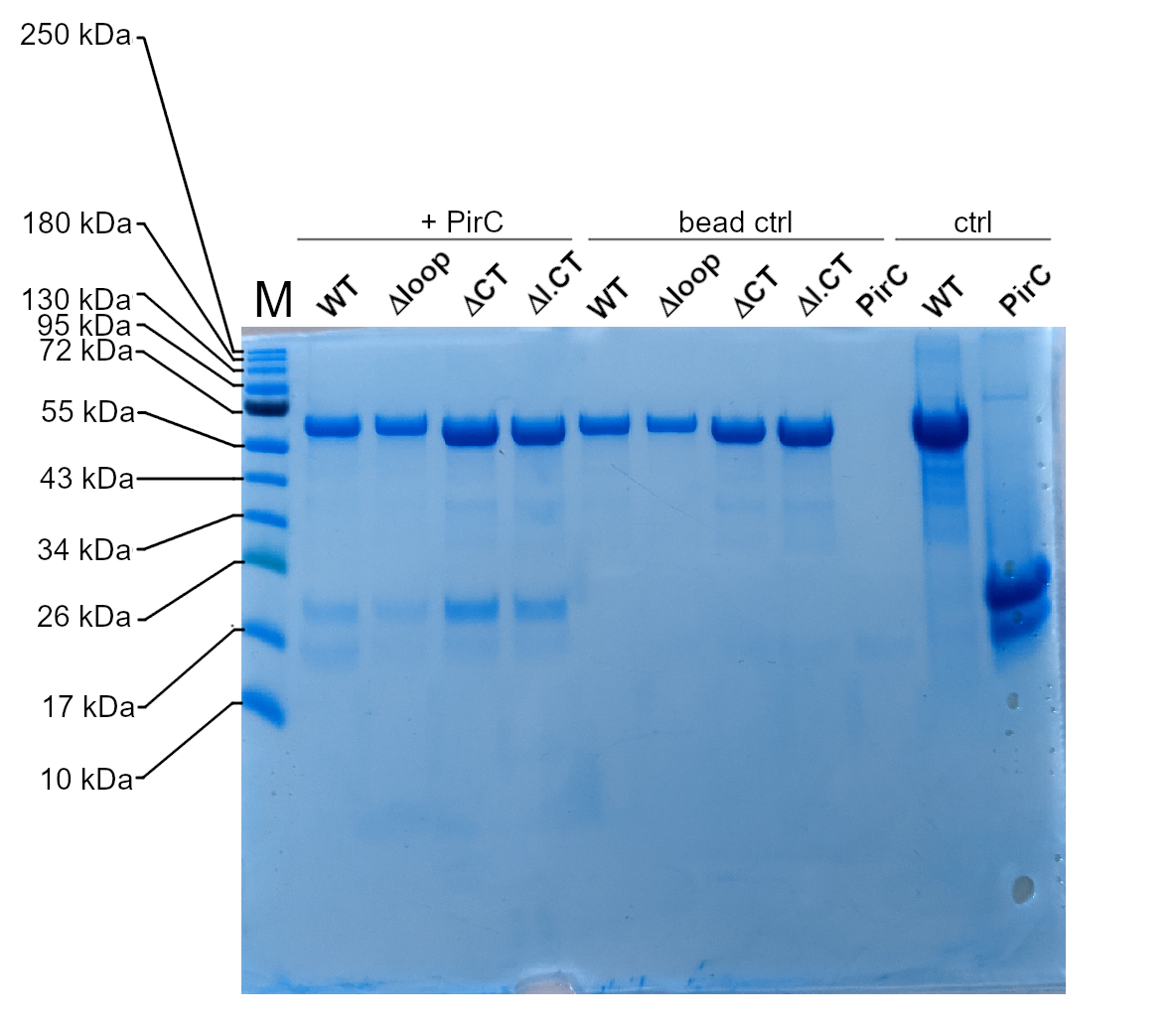


Figure S 5 – Pulldown assay of immobilised strep-PGAM variants on strep-tactin magnetic beads with His-Tagged PirC. The four columns after the standard band M are the samples, the flowing five columns are the different proteins without interaction incubation. The last two columns are the size control of PGAM and PirC.


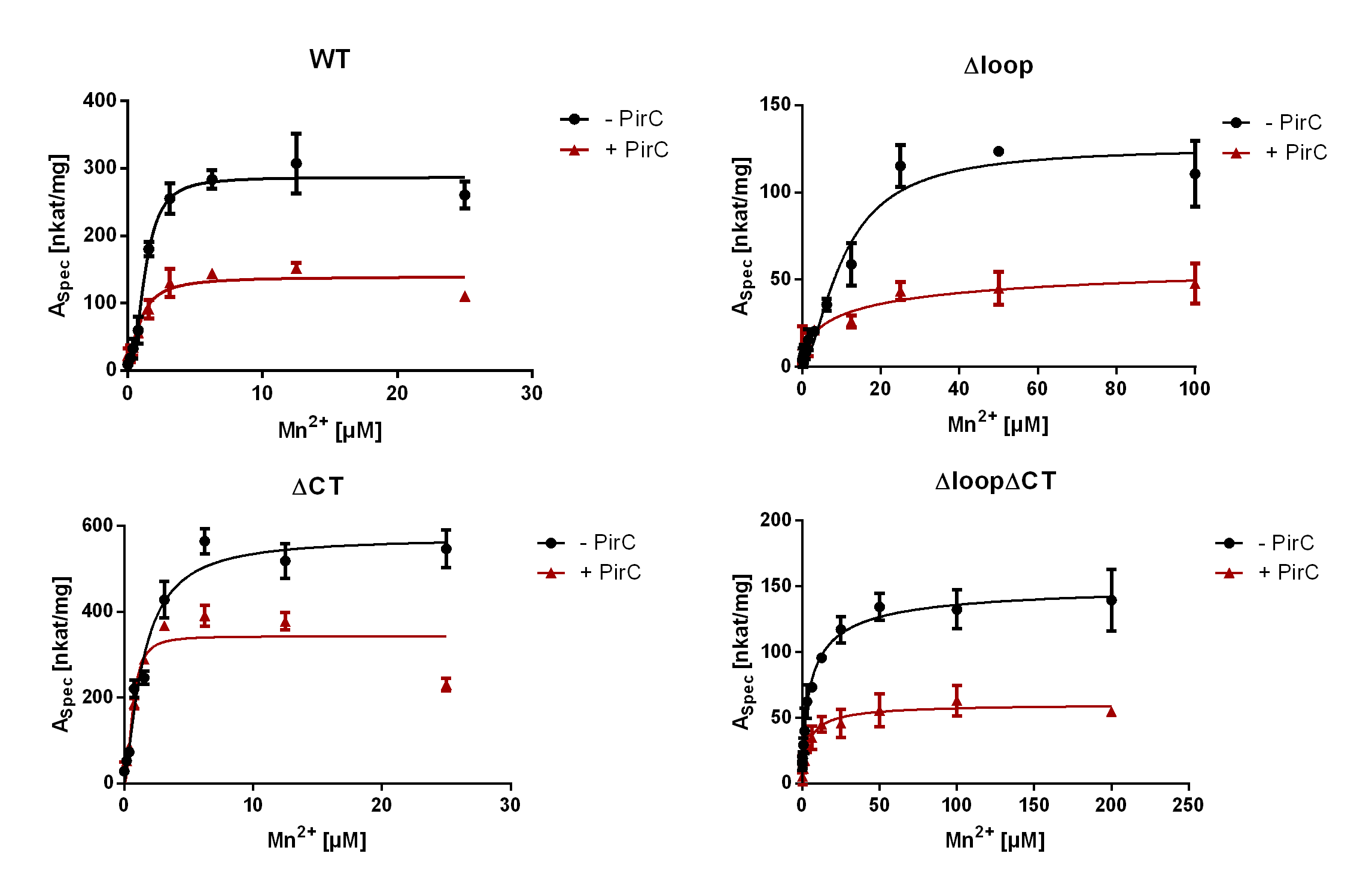


**iPGAM-**

**iPGAM-**

**iPGAM-**

**iPGAM-**

Figure S 6 – Effect of the deletion of the sub-structures on the activity of iPGAM and the inhibition with PirC in dependence of the manganese concentration (A) Hill kinetics of WT without (black) and with PirC inhibition (red) at a concentration of 1.5 mM 3-PGA. Hill coefficiencies: - PirC = 2.319 ± 0.336; + PirC = 1.498 ± 0.333. Khalf: - PirC = 1.268 ± 0.093 µM; +PirC = 0.880 ± 0.150 µM (B) Hill kinetics of Δloop without (black) and with PirC inhibition (red). Hill coefficiencies: - PirC = 1.605 ± 0.270; + PirC = 0.5798 ± 0,187, Khalf: - PirC = 10.54 ± 1.43 µM; +PirC = 0.13.75 ± 18.74µM (C) Hill kinetics of ΔCT without (black) and with PirC inhibition (red). Hill coefficiencies: - PirC = 1.377 ± 0.184; + PirC = 2.079 ± 0.537, Khalf: - PirC = 1.466 ± 0.175 µM; + PirC = 0.666 ± 0.095 µM (D) Hill kinetics of ΔloopΔCT without (black) and with PirC inhibition (red). Hill coefficiencies: - PirC = 0.728 ± 0.091; + PirC = 0.854 ± 0.181, Khalf: - PirC = 5.781 ± 1.462 µM; +PirC = 4.037 ± 1.300 µM. Each point represents the main of three independent technical replicates. The error bar depicts the standard deviation of the triplicates.


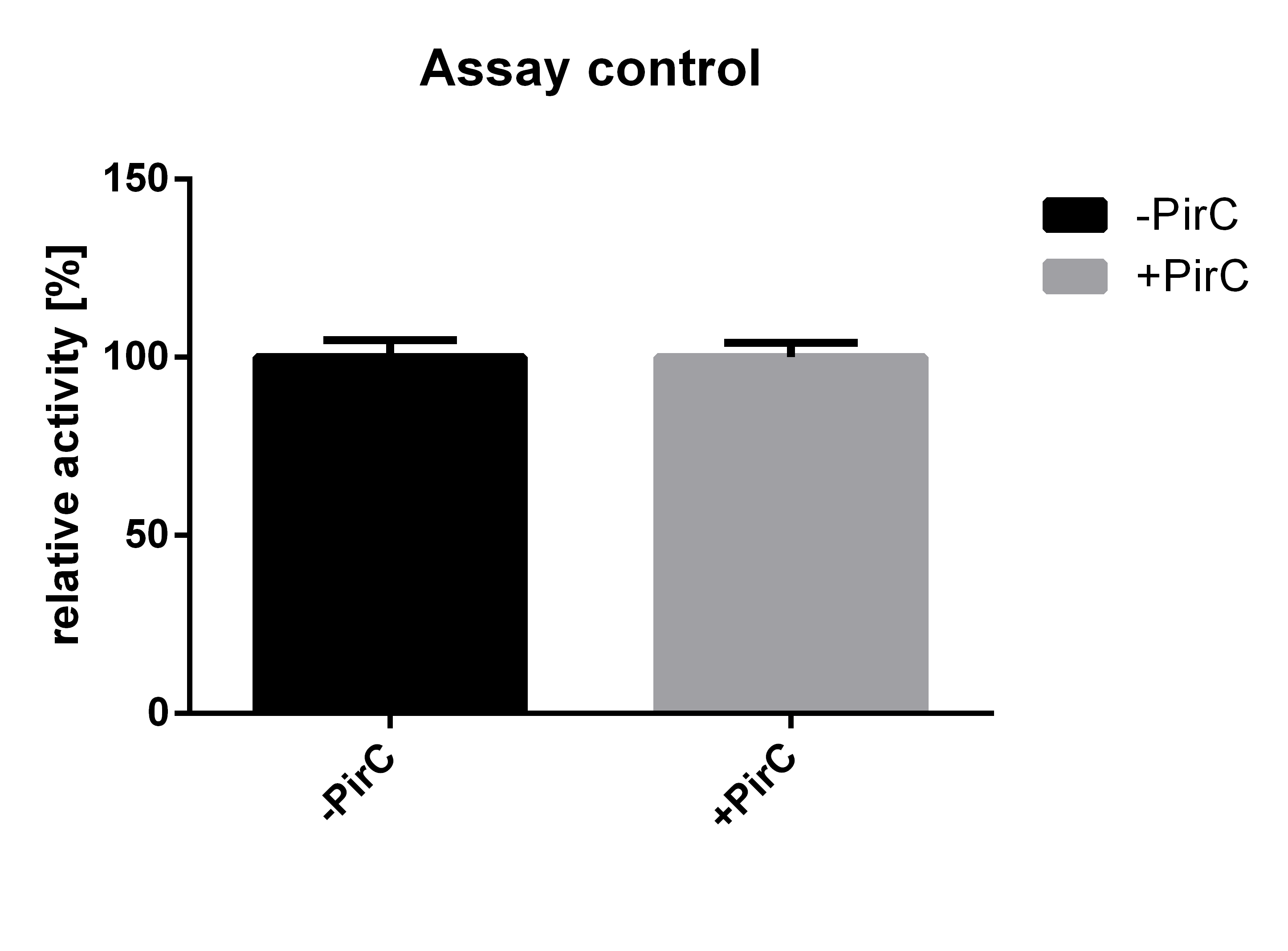


**E**


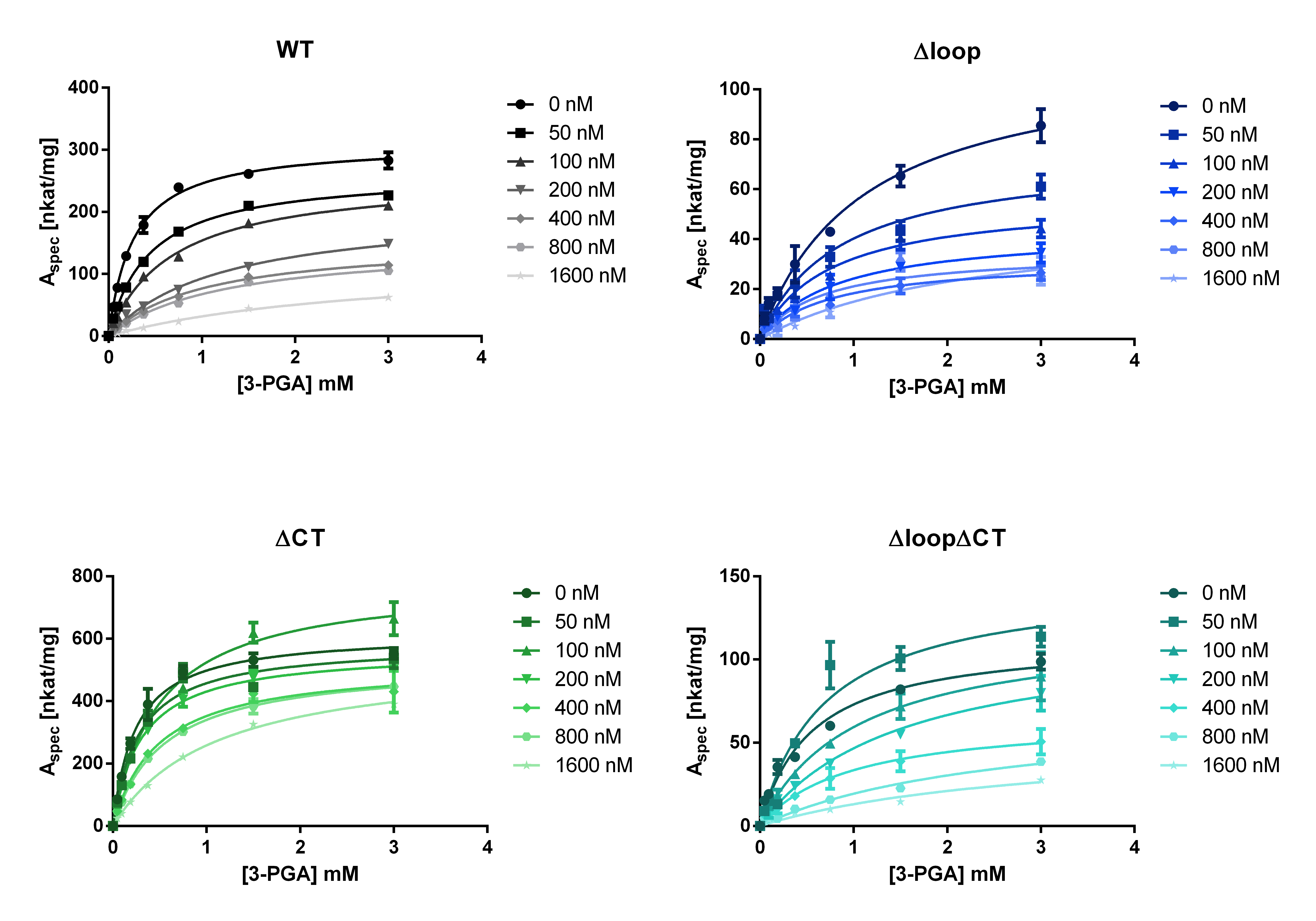


**iPGAM-**

**iPGAM-**

**iPGAM-**

**iPGAM-**

Figure S 7 – Effect of PirC on the PGAM variants in varying concentrations of PirC. (A) Michaelis-Menten kinetics of PGAM-WT (B) Michaelis-Menten kinetics of PGAM-Δloop. (C) Michaelis-Menten kinetics of PGAM-ΔCT (D) Michaelis-Menten kinetics of PGAM-ΔloopΔCT . Each points represents the main of three independent technical replicates. The error bar depicts the standard deviation of the triplicates. (E) Control of PGAM assay components by adding 2-PGA instead of 3-PGA and 1600 nM PirC.

**Supp.Figure 9** – Inhibtion mechanism and effect of PirC on the PGAM variants in varying concentrations of PirC. (A) Hanes-Woolf kinetics of PGAM_WT_ (B) Hanes-Woolf kinetics of PGAM_Δloop._ (C) Hanes-Woolf kinetics of PGAM_ΔCT_ (D) Hanes-Woolf kinetics of PGAM_ΔloopΔCT . ._Each points represents the main of three independent technical replicates. The error bar depicts the standard deviation of the triplicates.

4


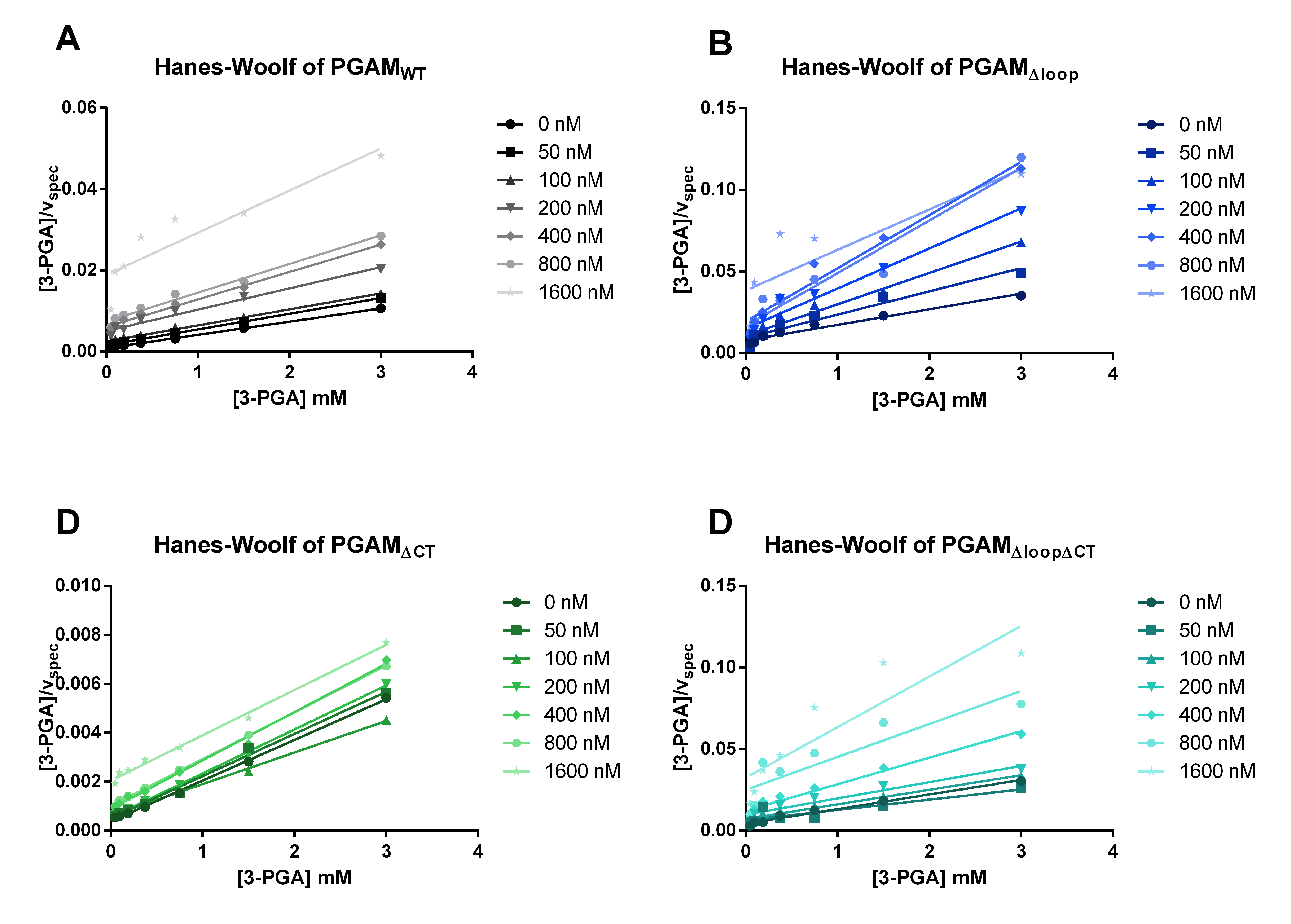


Figure S 8 – Inhibition mechanism and effect of PirC on the PGAM variants in varying concentrations of PirC. (A) Hanes-Woolf kinetics of PGAM-WT(B) Hanes-Woolf kinetics of PGAM-Δloop_._ (C) Hanes-Woolf kinetics of PGAM-ΔCT (D) Hanes-Woolf kinetics of PGAM-ΔloopΔCT _._ Each point represents the main of three independent technical replicates. The error bar depicts the standard deviation of the triplicates.

Table S 1 - Calculated kinetic parameters of all variants with all concentrations of PirC

## Supplemental Material & Methods

### Multiple alignments and phylogenetic tree calculation of phosphoglycerate mutases

The alignments were done with Matlab® using the functions multialign in the following script. This function performs a progressive multiple alignment computed after pairwise alignment. A Blosum64 matrix was used for scoring. The phylogenetic tree based on this alignment was performed with IQ-tree web server developed by Trifinopoulos et al. 2016 (1). This software calculates the tree with the maximum likelihood method. The Tree calculation was done again with a Blosum64 scoring matrix and an ultrafast bootstrap branch analysis with 1000 alignments.

The resulting tree was visualized with *iTol,* which was developed and is provide by The EMBL(European Molecular Biology Laboratory) Heidelberg (2).

### SWISS-MODEL structure prediction

The structure of the iPGAM was predicted using the SWISS-Model workspace developed by the working group of Thorsten Schwede and published in 2018. SWISS-Model predicts structures by homology modelling against known structures and is provided by the Swiss Institute of Bioinformatics (Lausanne, Switzerland) (3, 4).

### AlphaFold Prediction

The structures were also predicted using the AlphaFoldServer provided by Google DeepMind (London, UK). It uses the algorithm of AlphaFold 3 developed by Abramson et al. (2024).(6).

The accuracy of the AlphaFold prediction is depicted via three different parameters.

1. **pLDDT** (predicted Local Distance Difference Test)- superpositions-free score ranging from zero to 100. High confidence is indicated via values higher than 80 and values below 50 indicate low confidence.
2. **PAE** (Predicted Aligned Error) – graphical display of orientation and relative position. Dark = low orientation and relative position error; light = high orientation and relative position error.
3. **pTM & ipTM** (predicted template modelling & interface predicted template modelling) – derived values from template modelling score described by Xu et al. 2010 (7). Measures of overall accuracy of the individual proteins in a prediction of a complex (pTM) and accuracy of the whole complex (ipTM).

The confidence values are described in more detail in the “Frequently Asked Questions” section of the AlphaFold server.

(<https://alphafoldserver.com/faq#how-can-i-interpret-confidence-metrics-to-check-the-accuracy-of-structures>)

### Molecular Cloning, mutagenesis and Plasmids

Gibson Assembly (GA) and mutagenesis PCR were used to create plasmids. The GA was done according to the manufacturer protocol (NEB E2611S/L, E5510S) using a master mix of T5-Exonucleases (0.08 Units), Q5® High-fidelity DNA-Polymerase (0.5 Units) and Taq DNA Ligase (80 Units) (New England Biolabs, Boston) in Isobuffer (pH 7.5, 100 mM Tris, 1 mM MgCl2, 1 mM DTT, 0.2 mM dNTP’s, 1 mM NAD, and 25 % PEG-8000. The Assembly Fragments were linearized via PCR. To perform the assembly, 5 µl of an Assembly mixture was mixt with the master mix and incubated for 60 min at 50 °C.

According to the manufacturer protocol, iPGAM was mutated with the Q5® Site-Directed Mutagenesis Kit (NEB, E0554). Chemical-competent *E. coli* Stellar cells were used for the transformation.

Plasmids created and used in this study are listed in Table 3.

Table S 2 – List of used plasmids in this study

| **plasmid** | **Purpose** | **Source** |
| --- | --- | --- |
| pTO104 | Replacement of iPGAM gene (*slr1945*) by mutated iPGAM gene (iPGAM-Δloop) and Spec^R^ | This study |
| pTO106 | Replacement of iPGAM gene (*slr1945*) by mutated iPGAM gene (iPGAM-ΔCT) and Cm^R^ | This study |
| pTO107 | Replacement ofiPGAM gene (*slr1945*) by mutated iPGAM gene (iPGAMΔloopΔCT) and Cm^R^ | This study |
| pJA1 | Deletion of pirC-gene (*sll0944*) via Spec^R^ cassette | This study |
| pJS15 | Expression of strep-tagged P_II_ protein (Ssl0707) in *E. coli* | [NO_PRINTED_FORM] (8) |
| pJS22 | Expression of His8-Tagged PirC (Sll0944) in *E. coli* T7-strains | [NO_PRINTED_FORM] (9) |
| pJS26 | Expression of His8-Tagged P_II_ protein (Ssl0707) in *E. coli* T7-strains | [NO_PRINTED_FORM] (8) |
| pJS27 | Expression of strep-tagged PirC (Sll0944) in *E. coli* | [NO_PRINTED_FORM] (9) |
| pET-28a(+)-iPGAM | Expression His8-tagged 222iPGAM (Slr1945) in *E. coli* T7-strains | [NO_PRINTED_FORM] (9) |
| pTO301 | Expression of His8-tagged iPGAMΔloop in *E. coli* | This study |
| pTO304 | Expression of strep-tagged iPGAM in *E. coli* | This study |
| pTO305 | Expression of strep-tagged iPGAMΔloop in *E. coli* | This study |
| pTO307 | Expression of strep-tagged iPGAMΔCT in *E. coli* | This study |
| pTO308 | Expression of strep-tagged iPGAMΔloopΔCT in *E. coli* | This study |

### Strains

The strains used in this study are listed in Table 4.

Table S 3 – List of used organisms in this study

| **Organism** | **Strain** | **Genotype** | **Purpose** |
| --- | --- | --- | --- |
| *E. coli* | NEB10β | Δ(ara-leu) 7697 araD139 fhuA ΔlacX74 galK16 galE15 e14- Φ80dlacZΔM15 recA1 relA1 endA1 nupG rpsL (StrR) rph spoT1 Δ(mrr-hsdRMS-mcrBC) | Molecular Cloning |
| *E. coli* | Lemo21(DE3) | fhuA2 [lon] ompT gal (λ DE3) [dcm] ΔhsdS/ pLemo(CamR)  λ DE3 = λ sBamHIo ΔEcoRI-B int::(lacI::PlacUV5::T7 gene1) i21 Δnin5  pLemo = pACYC184-PrhaBAD-lysY | Protein Expression |
| *Synechocystis* sp. PCC 6803 | wildtype glucose-sensitive | WT | Background strain, control |
| *Synechocystis* sp. PCC 6803 | ΔPirC | *sll0944*::Spec^R^ | Control strain (This study) |
| *Synechocystis* sp. PCC 6803 | iPGAM-Δloop | iPGAM::PGAMΔloop-Spec^R^ | iPGAM variant testing (This study) |
| *Synechocystis* sp. PCC 6803 | iPGAM-ΔCT | iPGAM::iPGAMΔCT-Cm^R^ | iPGAM variant testing (This study) |
| *Synechocystis* sp. PCC 6803 | iPGAM-ΔloopΔCT | iPGAM::iPGAMΔloop ΔCT-Spec^R^ | iPGAM variant testing (This study) |

### Cultivation of Cyanobacteria

Growth experiments and precultures of *Synechocystis* were cultivated in BG_11_ with the composition explained by (34). Baffle-free 200 ml Erlenmeyer flasks were used for 50 ml cultures. Standard cultivation was performed at 28 °C with continuous shaking at 125 rpm, either at constant (24 h · d^–1^, ∼50 μE · m^‑2^ · s^–1^) or fluctuating (12 h light; ∼50 μE m^–2^ · s^–1^/12 h dark) illumination. For different experiments, the BG_11_ was adjusted as listed in Table 3. When necessary, appropriate antibiotics were supplemented in the media to ensure the continuity of the mutation. The growth was recorded by measuring the OD_750._

Table S 4 – Exchanges in media composition for different purposes

| Type of Experiment | Adjustment |
| --- | --- |
| Control | no adjustment |
| Ammonium growth | Replacement of NaNO_3_ to 5 mM Ammonium chloride. The lost Na+ ions were re-supplemented by 17 mM NaCl. (BG_11_, _Ammonia_) |
| Nitrogen depletion | Removal of NaNO_3_ and supplementation of 17 mM NaCl. BG_11,_0 |

For nitrogen deficiency experiments, pre-cultures of *Synechocystis* were cultivated for three days, as described previously, at an initial OD750 of 0.1. Experimental cultures were prepared in BG_11_ medium with a set starting OD750 of 0.2 and grown for two days under identical conditions until they reached an OD750 of 0.6-1. For the nitrogen depletion experiments, cells from the cultures were harvested by centrifugation (4000 g, 10 min), washed with and resuspended in BG_11,_0 medium to create cultures with an initial OD750 of 0.4.

For ammonium experiments, cells were diluted in fresh BG_11_, _Ammonia_ medium and adjusted to an OD750 of 0.2.

Escherichia coli cultures were grown on LB medium and agar. Lennox broth: 5 g · l^-1^ Yeast extract, 10 g · l^-1^ Tryptone, NaCl 5 g · l^-1^, and solid: 15 g · l^-1^ agar were used.

### Expression and Purification of Proteins

*E. coli* Lemo21(DE3) was used for the overexpression of the various kinds of proteins. His-tagged proteins were expressed as described in the manufactured expression protocol in 2-fold concentrated LB media with appropriate antibiotics. An overnight expression was induced by adding 400 μM IPTG at 20 °C during continuous shaking at 120 rpm. The expression of strep-tagged proteins based on pASK-Iba5Plus expression plasmid was induced by adding 200 μg·l^-1^ anhydrotetracycline.

The heterologous proteins containing His-tags were purified via 5 ml Ni-NTA HisTrap columns (Cytiva, Marlborough, USA). The cells were lysed in 50 ml lysis buffer containing 50 mM Tris/HCl buffer pH 8, 300 ml NaCl, 1 mM DTT, and cOmplete™ (Roche, Basel, Switzerland). The His-tagged proteins were loaded on the Ni-NTA column with Buffer A containing 50 mM Tris/HCl pH 8.0, 300 ml NaCl and eluted via a gradient of increasing imidazole (0-500 mM, Buffer B) using a ÄKTAPurifier™ System (Cytiva, Marlborough, USA). After this first purification, the proteins were purified further via size exclusion chromatography using a Superdex™ 200 Increase 10/300 GL (Cytiva, Marlborough, USA) with 50 mM Tris/HCl buffer containing 100 mM KCl and 0.5 mM EDTA.

5 ml Strep-tactin® superflow columns (IBA Lifescience, Göttingen, Germany) were used to purify Strep-tagged proteins. Cells were lysed in lysis buffer containing 100 mM Tris/HCl pH 8, 150 mM NaCl, and cOmplete™ (Roche, Basel, Switzerland). The proteins were loaded on the column and eluted with a buffer containing 2.5 mM Desthiobiotin. The buffer of each purified protein was exchanged via dialysis using dialysis buffer (50 mM Tris/HCl pH 8, 100 mm KCl, 40 % glycerol) and a 3 kDa cutoff dialysis tube. According to previous studies, all purification steps were checked via SDS-PAGE.

### Mass photometry using the Refyn OneMP

A mass photometry experiment was used to study the variants' oligomerization and the stoichiometry of the iPGAM-PirC complex. The meaWu & Piszczek (2021).ording to Wu & Piszczek (2021). All experiments were performed in a buffer containing 25 mM Tris-HCl, 50 mM KCl and 50 µM MnCl_2_ at pH 8. The buffer and all samples were filtered through a 0.22 µM sterile filter, and the buffer was degassed by vacuum degassing. As coverslips, the High-Precision Microscope Cover Glasses (Marienfeld, Lauda-Königshofen, Germany) were used, which were cleaned three times in ultrapure water and 100 % isopropanol under a sterile hood. The coverslips were adjusted onto the objective, and the CultureWell™ Reusable Gasket was placed on it. The machine was focused by adding 10 µl of the buffer in the gaskets on the coverslips. Afterwards, 10 µl of the sample was added to the buffer and measured for 1 min. The Refyn OneMP was calibrated using ovalbumin (43 kDa, Hen egg), cobalamin (75 kDa, chicken egg white), and aldolase (157 kDa, rabbit muscle) from the Gel Filtration Calibration Kit LMW (Cytiva, Marlborough, USA). The data was analyzed using the DiscoverMP® software.

### BLI using the Octet K2 System

*In vitro* binding studies were done using bio-layer interferometry (BLI) using the Octet K2 system (Sartorius, Göttingen, Germany) according to the Bio-protocol (12). The experiments were performed in HEPES buffer (20 mM HEPES-KOH pH 8.0, 50 µM MnCl2, 0.005 % NP-40, In the first step, His6-iPGAM (1000 nM) were immobilized on Ni-NTA sensors (Satorius), followed by a 60 sec baseline measurement. For the binding of PirC, the biosensors were dipped into the PirC solution for 180-sec (Association), with concentrations ranging between 9.375 nM – 1500 nM. A 300-sec dissociation step terminated the assay. One measurement without any interaction partner was performed to prevent false positive results in each experimental set. The biosensors were regenerated after each use with 10 mM glycine (pH 1.7) and 10 mM NiCl_2_, as proposed in manufacturer recommendations. The recorded curves of a set were preprocessed by aligning them to the average of the baseline step and the dissociation step. The response in equilibrium (Req) was calculated using the Data Analysis Software of the Octet System. The Concentration versus Req plots were made.

### Phosphoglycerate Mutase Assay

The iPGAM activity was determined by a coupled enzyme assay as adapted as described previously (9). Around 0.6 μg of purified iPGAM was used in a 200 µl reaction. The reaction mixture containing 20 mM HEPES-KOH (pH 8,0), 100 mM KCl, 5 mM MgSO4, 0.2 mM MnCl2, 50 μg ml^-1^ BSA, 1 mM DTT, 0.4  mM  ADP, 0.2  mM  NADH, 0.1  U  enolase (Sigma Aldrich, St. Louis, USA), 0.4  U  Pyruvate kinase (Roche, Basel, Switzerland), 0.4 U Lactate dehydrogenase (Roche, Basel, Switzerland) and 1 μg iPGAM was pre-warmed to 30 °C. The Assay was started by adding the 3-PGA solutions. The resulting decrease of NADH over time was recorded in a 96-well plate using Bioetek Epoch2 (Agilent, Santa Clara) at 340 nm. A blank assay without 3-PGA was also performed, and no decrease was detectable.(13)

### Glycogen Measurement

The glycogen content was quantified according to previous studies from 2 ml *Synechocystis* culture samples (14). The glycogen was hydrolyzed to glucose with 4,4 U · μl-1 amyloglucosidase from *Aspergillus niger* (Sigma Aldrich, St. Louis, USA) for 2 h at 60 °C. The resulting glucose concentration was measured via an o-toluidine assay (15).The samples were boiled in 1:6 dilution with a 6 % o-toluidine reagent (in glacial acetic acid) for 10 min, then cooled on ice and measured with Spark M10 (Tecan, Männedorf, Switzerland) at 635 nm. The concentration of samples was calculated using a calibration curve of a defined quantity of glucose (0, 10 μg, 50 μg, 100 μg, 250 μg, and 500 μg).

### PHB Quantification

As described previously, polyhydroxybutyrate (PHB) was detected using high-performance liquid chromatography (HPLC) (9, 16, 17).

## References

1. J. Trifinopoulos, L. T. Nguyen, A. von Haeseler, B. Q. Minh, W-IQ-TREE: a fast online phylogenetic tool for maximum likelihood analysis. *Nucleic Acids Res* **44**, W232–W235 (2016).

2. I. Letunic, P. Bork, Interactive Tree of Life (iTOL) v6: recent updates to the phylogenetic tree display and annotation tool. *Nucleic Acids Res* **52**, W78–W82 (2024).

3. N. Guex, M. C. Peitsch, T. Schwede, Automated comparative protein structure modeling with SWISS-MODEL and Swiss-PdbViewer: A historical perspective. *Electrophoresis* **30**, S162–S173 (2009).

4. A. Waterhouse, *et al.*, SWISS-MODEL: homology modelling of protein structures and complexes. *Nucleic Acids Res* **46**, W296 (2018).

5. J. Abramson, *et al.*, Accurate structure prediction of biomolecular interactions with AlphaFold 3. *Nature 2024 630:8016* **630**, 493–500 (2024).

6. J. Abramson, *et al.*, Accurate structure prediction of biomolecular interactions with AlphaFold 3. *Nature 2024 630:8016* **630**, 493–500 (2024).

7. J. Xu, Y. Zhang, How significant is a protein structure similarity with TM-score = 0.5? *Bioinformatics* **26**, 889–895 (2010).

8. J. Scholl, L. Dengler, L. Bader, K. Forchhammer, Phosphoenolpyruvate carboxylase from the cyanobacterium Synechocystis sp. PCC 6803 is under global metabolic control by PII signaling. *Mol Microbiol* **114**, 292–307 (2020).

9. T. Orthwein, *et al.*, The novel PII-interactor PirC identifies phosphoglycerate mutase as key control point of carbon storage metabolism in cyanobacteria. *Proc Natl Acad Sci U S A* **118**, e2019988118 (2021).

10. M. Mager, *et al.*, Interlaboratory Reproducibility in Growth and Reporter Expression in the Cyanobacterium Synechocystis sp. PCC 6803. *ACS Synth Biol* **12**, 1823–1835 (2023).

11. D. Wu, G. Piszczek, Standard protocol for mass photometry experiments. *European Biophysics Journal* **50**, 403–409 (2021).

12. T. Orthwein, L. F. Huergo, K. Forchhammer, K. A. Selim, Kinetic analysis of a protein-protein complex to determine its dissociation constant (kd) and the effective concentration (ec50) of an interplaying effector molecule using bio-layer interferometry. *Bio Protoc* **11** (2021).

13. Y. H. Huang, *et al.*, Structure-Based Mechanisms of a Molecular RNA Polymerase/Chaperone Machine Required for Ribosome Biosynthesis. *Mol Cell* **79**, 1024-1036.e5 (2020).

14. S. Doello, A. Klotz, A. Makowka, K. Gutekunst, K. Forchhammer, A Specific Glycogen Mobilization Strategy Enables Rapid Awakening of Dormant Cyanobacteria from Chlorosis. *Plant Physiol* **177**, 594–603 (2018).

15. K. M. DUBOWSKI, An o-Toluidine Method for Body-Fluid Glucose Determination. *Clin Chem* **8**, 215–235 (1962).

16. M. Koch, K. W. Berendzen, K. Forchhammer, On the Role and Production of Polyhydroxybutyrate (PHB) in the Cyanobacterium Synechocystis sp. PCC 6803. *Life* **10** (2020).

17. M. Koch, *et al.*, Maximizing PHB content in Synechocystis sp. PCC 6803: a new metabolic engineering strategy based on the regulator PirC. *Microb Cell Fact* **19**, 1–12 (2020).
